# Integrated analysis of environmental and genetic influences on cord blood DNA methylation in new-borns

**DOI:** 10.1038/s41467-019-10461-0

**Published:** 2019-06-11

**Authors:** Darina Czamara, Gökçen Eraslan, Christian M. Page, Jari Lahti, Marius Lahti-Pulkkinen, Esa Hämäläinen, Eero Kajantie, Hannele Laivuori, Pia M. Villa, Rebecca M. Reynolds, Wenche Nystad, Siri E. Håberg, Stephanie J. London, Kieran J. O’Donnell, Elika Garg, Michael J. Meaney, Sonja Entringer, Pathik D. Wadhwa, Claudia Buss, Meaghan J. Jones, David T. S. Lin, Julie L. MacIsaac, Michael S. Kobor, Nastassja Koen, Heather J. Zar, Karestan C. Koenen, Shareefa Dalvie, Dan J. Stein, Ivan Kondofersky, Nikola S. Müller, Fabian J. Theis, Major Depressive Disorder Working Group of the Psychiatric Genomics Consortium, Naomi R. Wray, Stephan Ripke, Manuel Mattheisen, Maciej Trzaskowski, Enda M. Byrne, Abdel Abdellaoui, Mark J. Adams, Esben Agerbo, Tracy M. Air, Till F. M. Andlauer, Silviu-Alin Bacanu, Marie Bækvad-Hansen, Aartjan T. F. Beekman, Tim B. Bigdeli, Douglas H. R. Blackwood, Julien Bryois, Henriette N. Buttenschøn, Jonas Bybjerg-Grauholm, Na Cai, Enrique Castelao, Jane Hvarregaard Christensen, Toni-Kim Clarke, Jonathan R. I. Coleman, Lucía Colodro-Conde, Baptiste Couvy-Duchesne, Nick Craddock, Gregory E. Crawford, Gail Davies, Ian J. Deary, Franziska Degenhardt, Eske M. Derks, Nese Direk, Conor V. Dolan, Erin C. Dunn, Thalia C. Eley, Valentina Escott-Price, Farnush Farhadi Hassan Kiadeh, Hilary K. Finucane, Andreas J. Forstner, Josef Frank, Héléna A. Gaspar, Michael Gill, Fernando S. Goes, Scott D. Gordon, Jakob Grove, Lynsey S. Hall, Christine Søholm Hansen, Thomas F. Hansen, Stefan Herms, Ian B. Hickie, Per Hoffmann, Georg Homuth, Carsten Horn, Jouke-Jan Hottenga, David M. Hougaard, Marcus Ising, Rick Jansen, Eric Jorgenson, James A. Knowles, Isaac S. Kohane, Julia Kraft, Warren W. Kretzschmar, Jesper Krogh, Zoltán Kutalik, Yihan Li, Penelope A. Lind, Donald J. MacIntyre, Dean F. MacKinnon, Robert M. Maier, Wolfgang Maier, Jonathan Marchini, Hamdi Mbarek, Patrick McGrath, Peter McGuffin, Sarah E. Medland, Divya Mehta, Christel M. Middeldorp, Evelin Mihailov, Yuri Milaneschi, Lili Milani, Francis M. Mondimore, Grant W. Montgomery, Sara Mostafavi, Niamh Mullins, Matthias Nauck, Bernard Ng, Michel G. Nivard, Dale R. Nyholt, Paul F. O’Reilly, Hogni Oskarsson, Michael J. Owen, Jodie N. Painter, Carsten Bøcker Pedersen, Marianne Giørtz Pedersen, Roseann E. Peterson, Erik Pettersson, Wouter J. Peyrot, Giorgio Pistis, Danielle Posthuma, Jorge A. Quiroz, Per Qvist, John P. Rice, Brien P. Riley, Margarita Rivera, Saira Saeed Mirza, Robert Schoevers, Eva C. Schulte, Ling Shen, Jianxin Shi, Stanley I. Shyn, Engilbert Sigurdsson, Grant C. B. Sinnamon, Johannes H. Smit, Daniel J. Smith, Hreinn Stefansson, Stacy Steinberg, Fabian Streit, Jana Strohmaier, Katherine E. Tansey, Henning Teismann, Alexander Teumer, Wesley Thompson, Pippa A. Thomson, Thorgeir E. Thorgeirsson, Matthew Traylor, Jens Treutlein, Vassily Trubetskoy, André G. Uitterlinden, Daniel Umbricht, Sandra Van der Auwera, Albert M. van Hemert, Alexander Viktorin, Peter M. Visscher, Yunpeng Wang, Bradley T. Webb, Shantel Marie Weinsheimer, Jürgen Wellmann, Gonneke Willemsen, Stephanie H. Witt, Yang Wu, Hualin S. Xi, Jian Yang, Futao Zhang, Volker Arolt, Bernhard T. Baune, Klaus Berger, Dorret I. Boomsma, Sven Cichon, Udo Dannlowski, E. J. C. de Geus, J. Raymond DePaulo, Enrico Domenici, Katharina Domschke, Tõnu Esko, Hans J. Grabe, Steven P. Hamilton, Caroline Hayward, Andrew C. Heath, Kenneth S. Kendler, Stefan Kloiber, Glyn Lewis, Qingqin S. Li, Susanne Lucae, Pamela A. F. Madden, Patrik K. Magnusson, Nicholas G. Martin, Andrew M. McIntosh, Andres Metspalu, Ole Mors, Preben Bo Mortensen, Bertram Müller-Myhsok, Merete Nordentoft, Markus M. Nöthen, Michael C. O’Donovan, Sara A. Paciga, Nancy L. Pedersen, Brenda W. J. H. Penninx, Roy H. Perlis, David J. Porteous, James B. Potash, Martin Preisig, Marcella Rietschel, Catherine Schaefer, Thomas G. Schulze, Jordan W. Smoller, Kari Stefansson, Henning Tiemeier, Rudolf Uher, Henry Völzke, Myrna M. Weissman, Thomas Werge, Cathryn M. Lewis, Douglas F. Levinson, Gerome Breen, Anders D. Børglum, Patrick F. Sullivan, Katri Räikkönen, Elisabeth B. Binder

**Affiliations:** 1 Max-Planck-Institute of Psychiatry, Department of Translational Research in Psychiatry, Munich, 80804 Germany; 2 Institute of Computational Biology, Helmholtz-Zentrum München, German Research Center for Environmental Health, Neuherberg, 85764 Germany; 3 School of Life Sciences, Weihenstephan, Technische Universität München, Freising, 85354 Germany; 4 Oslo Centre for Biostatistics and Epidemiology, Research Support Unit, Oslo University Hospital, Oslo, 0372 Norway; 5 Center for Fertility and Health, Norwegian Institute of Public Health, Oslo, 0213 Norway; 6 Department of Psychology and Logopedics, Faculty of Medicine, University of Helsinki, Helsinki, 00014 Finland; 7 Helsinki Collegium for Advanced Studies, University of Helsinki, Helsinki, 00101 Finland; 8 British Heart Foundation Centre for Cardiovascular Science, Queen’s Medical Research Institute, University of Edinburgh, Edinburgh, EH16 4TJ UK; 9 HUSLAB and Department of Clinical Chemistry, Helsinki University, Helsinki, 00290 Finland; 10 Oulu University Hospital and University of Oulu, PEDEGO Research Unit, MRC Oulu, 90014 Finland; 11 Hospital for Children and Adolescents, University of Helsinki and Helsinki University Hospital, Helsinki, 00029 Finland; 12 National Institute for Health and Welfare, Helsinki, 00271 Finland; 13 Medical and Clinical Genetics and Obstetrics and Gynaecology University of Helsinki and Helsinki University Central Hospital, Helsinki, 00014 Finland; 14 Institute for Molecular Medicine Finland, Helsinki Institute of Life Science, University of Helsinki, Helsinki, 00014 Finland; 15 Faculty of Medicine and Life Sciences, University of Tampere, Tampere, 33100 Finland; 16 Department of Obstetrics and Gynecology, Tampere University Hospital, Tampere, 33100 Finland; 17 Department of Chronic Diseases and Ageing, Norwegian Institute of Public Health, Oslo, 0213 Norway; 18 Epidemiology Branch, National Institute of Environmental Health Sciences, National Institutes of Health, U.S. Department of Health and Human Services, Research Triangle Park, North Carolina, 20814 USA; 19 Ludmer Centre for Neuroinformatics and Mental Health, Douglas Mental Health University Institute, McGill University, Montreal, H3A 2B4 QC Canada; 20 Sackler Program for Epigenetics and Psychobiology at McGill University, Montreal, H3A 0G4 QC Canada; 21 Singapore Institute for Clinical Sciences, Singapore, 117609 Singapore; 22 Charité – Universitätsmedizin Berlin, corporate member of Freie Universität Berlin, Humboldt-Universität zu Berlin, and Berlin Institute of Health (BIH), Institute of Medical Psychology, Berlin, 10117 Germany; 23 University of California, Irvine, Development, Health, and Disease Research Program, Orange, CA 92697 USA; 24 Department of Psychiatry and Human Behavior, Obstetrics and Gynecology, and Epidemiology, University of California, Irvine, School of Medicine, Irvine, CA 92697 USA; 25 Centre for Molecular Medicine and Therapeutics, Department of Medical Genetics, University of British Columbia and the BC Children’s Hospital Research Institute, Vancouver, V5Z 4H4 BC Canada; 26 Department of Psychiatry and Mental Health, University of Cape Town, Cape Town, 7925 South Africa; 27 South African Medical Research Council (SAMRC), Unit on Risk and Resilience in Mental Disorders, Cape Town, 7505 South Africa; 28 Department of Paediatrics & Child Health and SAMRC Unit on Child and Adolescent Health, University of Cape Town, Cape Town, 7505 South Africa; 29 Department of Epidemiology, Harvard T. H. Chan School of Public Health, Boston, MA 02115 USA; 30 Department of Mathematics, Technische Universität München, Munich, 85748 Germany; 31 Department of Psychiatry and Behavioral Sciences, Emory University School of Medicine, Atlanta, 30329 USA; 32 Institute for Molecular Bioscience, The University of Queensland, Brisbane, 4072 QLD Australia; 33 Queensland Brain Institute, The University of Queensland, Brisbane, 4072 QLD Australia; 34 Analytic and Translational Genetics Unit, Massachusetts General Hospital, Boston, MA 02114 USA; 35 Department of Psychiatry and Psychotherapy, Universitätsmedizin Berlin Campus Charité Mitte, Berlin, 14129 Germany; 36 Medical and Population Genetics, Broad Institute, Cambridge, MA 02142 USA; 37 Centre for Psychiatry Research, Department of Clinical Neuroscience, Karolinska Institutet, Stockholm, 17177 SE Sweden; 38 Department of Biomedicine, Aarhus University, Aarhus, 8000 Denmark; 39 iSEQ, Centre for Integrative Sequencing, Aarhus University, Aarhus, 8000 Denmark; 40 iPSYCH, The Lundbeck Foundation Initiative for Integrative Psychiatric Research, Aarhus, 8000 Denmark; 41 Department of Biological Psychology & EMGO+ Institute for Health and Care Research, Vrije Universiteit Amsterdam, Amsterdam, 1081 BT Netherlands; 42 Division of Psychiatry, University of Edinburgh, Edinburgh, EH10 5HF UK; 43 Centre for Integrated Register-based Research, Aarhus University, Aarhus, 8210 Denmark; 44 National Centre for Register-Based Research, Aarhus University, Aarhus, 8210 Denmark; 45 Discipline of Psychiatry, University of Adelaide, Adelaide, 5000 SA Australia; 46 Munich Cluster for Systems Neurology (SyNergy), Munich, 81377 Germany; 47 Department of Psychiatry, Virginia Commonwealth University, Richmond, VA 22903 USA; 48 Center for Neonatal Screening, Department for Congenital Disorders, Statens Serum Institut, Copenhagen, 2300 Denmark; 49 Department of Psychiatry, Vrije Universiteit Medical Center and GGZ inGeest, Amsterdam, 1081 NL Netherlands; 50 Virginia Institute for Psychiatric and Behavior Genetics, Richmond, VA 23298 USA; 51 Department of Medical Epidemiology and Biostatistics, Karolinska Institutet, Stockholm, 17177 SE Sweden; 52 Department of Clinical Medicine, Translational Neuropsychiatry Unit, Aarhus University, Aarhus, 8240 Denmark; 53 Human Genetics, Wellcome Trust Sanger Institute, Cambridge, CB10 1SA UK; 54 Statistical genomics and systems genetics, European Bioinformatics Institute (EMBL-EBI), Cambridge, CB10 1 SD UK; 55 Department of Psychiatry, University Hospital of Lausanne, Prilly, Vaud, 1004 Switzerland; 56 MRC Social Genetic and Developmental Psychiatry Centre, King’s College London, London, WC2R 2LS UK; 57 Genetics and Computational Biology, QIMR Berghofer Medical Research Institute, Herston, 4006 QLD Australia; 58 Centre for Advanced Imaging, The University of Queensland, Saint Lucia, 4072 QLD Australia; 59 Queensland Brain Institute, The University of Queensland, Saint Lucia, 4072 QLD Australia; 60 Psychological Medicine, Cardiff University, Cardiff, CF14 4XN UK; 61 Center for Genomic and Computational Biology, Duke University, Durham, NC 27705 USA; 62 Division of Medical Genetics, Department of Pediatrics, Duke University, Durham, NC 27708 USA; 63 Centre for Cognitive Ageing and Cognitive Epidemiology, University of Edinburgh, Edinburgh, EH8 9JZ UK; 64 Institute of Human Genetics, University of Bonn, Bonn, 53127 DE Germany; 65 Life & Brain Center, Department of Genomics, University of Bonn, Bonn, 53127 Germany; 66 Epidemiology, Erasmus MC, Rotterdam, 3015 Zuid-Holland Netherlands; 67 Psychiatry, Dokuz Eylul University School Of Medicine, Izmir, 35220 Turkey; 68 Department of Psychiatry, Massachusetts General Hospital, Boston, MA 02114 USA; 69 Psychiatric and Neurodevelopmental Genetics Unit (PNGU), Massachusetts General Hospital, Boston, MA 02114 USA; 70 Stanley Center for Psychiatric Research, Broad Institute, Cambridge, MA 02142 USA; 71 Neuroscience and Mental Health, Cardiff University, Cardiff, CF24 4HQ UK; 72 Bioinformatics, University of British Columbia, Vancouver, V5Z 4S6 BC Canada; 73 Department of Mathematics, Massachusetts Institute of Technology, Cambridge, MA 02142 USA; 74 Department of Psychiatry (UPK), University of Basel, Basel, 4002 Switzerland; 75 Human Genomics Research Group, Department of Biomedicine, University of Basel, Basel, 4031 Switzerland; 76 Department of Genetic Epidemiology in Psychiatry, Central Institute of Mental Health, Medical Faculty Mannheim, Heidelberg University, Mannheim, 68159 Baden-Württemberg Germany; 77 Department of Psychiatry, Trinity College Dublin, Dublin, 8 Ireland; 78 Psychiatry & Behavioral Sciences, Johns Hopkins University, Baltimore, MD 21287 USA; 79 Genetics and Computational Biology, QIMR Berghofer Medical Research Institute, Brisbane, 4006 QLD Australia; 80 Bioinformatics Research Centre, Aarhus University, Aarhus, 8000 Denmark; 81 Institute of Genetic Medicine, Newcastle University, Newcastle upon Tyne, NE1 3BZ England; 82 Danish Headache Centre, Department of Neurology, Rigshospitalet, Glostrup, 2600 Denmark; 83 Institute of Biological Psychiatry, Mental Health Center Sct. Hans, Mental Health Services Capital Region of Denmark, Copenhagen, 4000 Denmark; 84 iPSYCH, The Lundbeck Foundation Initiative for Psychiatric Research, Copenhagen, 8000 Denmark; 85 Brain and Mind Centre, University of Sydney, Sydney, 2050 NSW Australia; 86 Interfaculty Institute for Genetics and Functional Genomics, Department of Functional Genomics, University Medicine and Ernst Moritz Arndt University Greifswald, Greifswald, 17489 Mecklenburg-Vorpommern Germany; 87 Roche Pharmaceutical Research and Early Development, Pharmaceutical Sciences, Roche Innovation Center Basel, F. Hoffmann-La Roche Ltd, Basel, 4070 Switzerland; 88 Max Planck Institute of Psychiatry, Munich, 80804 Germany; 89 Division of Research, Kaiser Permanente Northern California, Oakland, CA 94612 USA; 90 Psychiatry & The Behavioral Sciences, University of Southern California, Los Angeles, CA 90033 USA; 91 Department of Biomedical Informatics, Harvard Medical School, Boston, MA 02115 USA; 92 Department of Medicine, Brigham and Women’s Hospital, Boston, MA 02115 USA; 93 Informatics Program, Boston Children’s Hospital, Boston, MA 02115 USA; 94 Wellcome Trust Centre for Human Genetics, University of Oxford, Oxford, OX3 7BN UK; 95 Department of Endocrinology at Herlev University Hospital, University of Copenhagen, Copenhagen, 2730 Denmark; 96 Institute of Social and Preventive Medicine (IUMSP), University Hospital of Lausanne, Lausanne, VD 1010 Switzerland; 97 Swiss Institute of Bioinformatics, Lausanne, VD 1015 Switzerland; 98 Division of Psychiatry, Centre for Clinical Brain Sciences, University of Edinburgh, Edinburgh, EH16 4SB UK; 99 Mental Health, NHS 24, Glasgow, G12 0XH UK; 100 Department of Psychiatry and Psychotherapy, University of Bonn, Bonn, 53105 Germany; 101 Statistics, University of Oxford, Oxford, OX1 3LB UK; 102 Psychiatry, Columbia University College of Physicians and Surgeons, New York, NY 10032 USA; 103 School of Psychology and Counseling, Queensland University of Technology, Brisbane, QLD 4059 Australia; 104 Child and Youth Mental Health Service, Children’s Health Queensland Hospital and Health Service, South Brisbane, QLD 4000 Australia; 105 Child Health Research Centre, University of Queensland, Brisbane, QLD 4101 Australia; 106 Estonian Genome Center, University of Tartu, Tartu, 51005 Estonia; 107 Medical Genetics, University of British Columbia, Vancouver, BC V6H 3N1 Canada; 108 Statistics, University of British Columbia, Vancouver, BC V6T 1Z4 Canada; 109 DZHK (German Centre for Cardiovascular Research), Partner Site Greifswald, University Medicine, University Medicine Greifswald, Greifswald, Mecklenburg-Vorpommern 17489 Germany; 110 Institute of Clinical Chemistry and Laboratory Medicine, University Medicine Greifswald, Greifswald, Mecklenburg-Vorpommern 17489 Germany; 111 Institute of Health and Biomedical Innovation, Queensland University of Technology, Brisbane, QLD 4059 Australia; 112 Humus, Reykjavik, 101 Iceland; 113 MRC Centre for Neuropsychiatric Genetics and Genomics, Cardiff University, Cardiff, CF24 4HQ UK; 114 Virginia Institute for Psychiatric & Behavioral Genetics, Virginia Commonwealth University, Richmond, VA 23298 USA; 115 Clinical Genetics, Vrije Universiteit Medical Center, Amsterdam, 1081HV Netherlands; 116 Complex Trait Genetics, Vrije Universiteit Amsterdam, Amsterdam, 1081 HV Netherlands; 117 Solid Biosciences, Boston, MA 02139 USA; 118 Department of Psychiatry, Washington University in Saint Louis School of Medicine, Saint Louis, MO 63110 USA; 119 Department of Biochemistry and Molecular Biology II, Institute of Neurosciences, Center for Biomedical Research, University of Granada, Granada, CP 18100 Spain; 120 Department of Psychiatry, University of Groningen, University Medical Center Groningen, Groningen, 9700 RB Netherlands; 121 Department of Psychiatry and Psychotherapy, Medical Center of the University of Munich, Campus Innenstadt, Munich, 80336 Germany; 122 Institute of Psychiatric Phenomics and Genomics (IPPG), Medical Center of the University of Munich, Campus Innenstadt, Munich, 80336 Germany; 123 Division of Cancer Epidemiology and Genetics, National Cancer Institute, Bethesda, MD 20892 USA; 124 Behavioral Health Services, Kaiser Permanente Washington, Seattle, WA 98112 USA; 125 Faculty of Medicine, Department of Psychiatry, University of Iceland, Reykjavik, 101 Iceland; 126 School of Medicine and Dentistry, James Cook University, Townsville, QLD 4811 Australia; 127 Institute of Health and Wellbeing, University of Glasgow, Glasgow, G12 8RZ UK; 128 deCODE Genetics/Amgen, Reykjavik, 101 Iceland; 129 College of Biomedical and Life Sciences, Cardiff University, Cardiff, CF14 4EP UK; 130 Institute of Epidemiology and Social Medicine, University of Münster, Münster, Nordrhein-Westfalen 48149 Germany; 131 Institute for Community Medicine, University Medicine Greifswald, Greifswald, Mecklenburg-Vorpommern 17489 Germany; 132 Department of Psychiatry, University of California, San Diego, San Diego, CA 92093 USA; 133 KG Jebsen Centre for Psychosis Research, Norway Division of Mental Health and Addiction, Oslo University Hospital, Oslo, 0407 Norway; 134 Clinical Neurosciences, University of Cambridge, Cambridge, CB2 1QW UK; 135 Internal Medicine, Erasmus MC, Rotterdam, Zuid-Holland 3015 Netherlands; 136 Roche Pharmaceutical Research and Early Development, Neuroscience, Ophthalmology and Rare Diseases Discovery & Translational Medicine Area, Roche Innovation Center Basel, F. Hoffmann-La Roche Ltd, Basel, 4070 Switzerland; 137 Department of Psychiatry and Psychotherapy, University Medicine Greifswald, Greifswald, Mecklenburg-Vorpommern 17475 Germany; 138 Department of Psychiatry, Leiden University Medical Center, Leiden, 2333 ZA Netherlands; 139 Virginia Institute of Psychiatric & Behavioral Genetics, Virginia Commonwealth University, Richmond, VA 23298 USA; 140 Computational Sciences Center of Emphasis, Pfizer Global Research and Development, Cambridge, MA 02139 USA; 141 Institute for Molecular Bioscience; Queensland Brain Institute, The University of Queensland, Brisbane, QLD 4072 Australia; 142 Department of Psychiatry, University of Münster, Münster, Nordrhein-Westfalen 48149 Germany; 143 Institute of Medical Genetics and Pathology, University Hospital Basel, University of Basel, Basel, 4031 Switzerland; 144 Institute of Neuroscience and Medicine (INM-1), Research Center Juelich, Juelich, 52425 Germany; 145 Amsterdam Public Health Institute, Vrije Universiteit Medical Center, Amsterdam, 1081 BT Netherlands; 146 Centre for Integrative Biology, Università degli Studi di Trento, Trento, Trentino-Alto Adige 38123 Italy; 147 Department of Psychiatry and Psychotherapy, Medical Center, University of Freiburg, Faculty of Medicine, University of Freiburg, Freiburg, 79104 Germany; 148 Psychiatry, Kaiser Permanente Northern California, San Francisco, CA 94115 USA; 149 Medical Research Council Human Genetics Unit, Institute of Genetics and Molecular Medicine, University of Edinburgh, Edinburgh, EH4 2XU UK; 150 Department of Psychiatry, University of Toronto, Toronto, ON M5T 1R8 Canada; 151 Centre for Addiction and Mental Health, Toronto, ON M6J 1H4 Canada; 152 Division of Psychiatry, University College London, London, W1T 7NF UK; 153 Neuroscience Therapeutic Area, Janssen Research and Development, LLC, Titusville, NJ 08560 USA; 154 Institute of Molecular and Cell Biology, University of Tartu, Tartu, 51010 Estonia; 155 Psychosis Research Unit, Aarhus University Hospital, Risskov, Aarhus, 8200 Denmark; 156 University of Liverpool, Liverpool, L69 3BX UK; 157 Mental Health Center Copenhagen, Copenhagen Universtity Hospital, Copenhagen, 2100 Denmark; 158 Human Genetics and Computational Biomedicine, Pfizer Global Research and Development, Groton, CT 06340 USA; 159 Psychiatry, Harvard Medical School, Boston, MA 02215 USA; 160 Medical Genetics Section, CGEM, IGMM, University of Edinburgh, Edinburgh, EH4 2XU UK; 161 Psychiatry, University of Iowa, Iowa City, IA 52246 USA; 162 Department of Psychiatry and Behavioral Sciences, Johns Hopkins University, Baltimore, MD 21287 USA; 163 Department of Psychiatry and Psychotherapy, University Medical Center Göttingen, Goettingen, Niedersachsen 37075 Germany; 164 Human Genetics Branch, NIMH Division of Intramural Research Programs, Bethesda, MD 20892-9663 USA; 165 Faculty of Medicine, University of Iceland, Reykjavik, 101 Iceland; 166 Child and Adolescent Psychiatry, Erasmus MC, Rotterdam, Zuid-Holland 3015 Netherlands; 167 Psychiatry, Erasmus MC, Rotterdam, Zuid-Holland 3015 Netherlands; 168 Psychiatry, Dalhousie University, Halifax, NS B3H 2E2 Canada; 169 Division of Epidemiology, New York State Psychiatric Institute, New York, NY 10032 USA; 170 Department of Clinical Medicine, University of Copenhagen, Copenhagen, 2200 Denmark; 171 Department of Medical & Molecular Genetics, King’s College London, London, WC2R 2LS UK; 172 Psychiatry & Behavioral Sciences, Stanford University, Stanford, CA 94305-5717 USA; 173 NIHR BRC for Mental Health, King’s College London, London, SE5 8AF UK; 174 Genetics, University of North Carolina at Chapel Hill, Chapel Hill, NC 27514 USA; 175 Psychiatry, University of North Carolina at Chapel Hill, Chapel Hill, NC 27514 USA

**Keywords:** DNA methylation, Epigenomics, Biomarkers

## Abstract

Epigenetic processes, including DNA methylation (DNAm), are among the mechanisms allowing integration of genetic and environmental factors to shape cellular function. While many studies have investigated either environmental or genetic contributions to DNAm, few have assessed their integrated effects. Here we examine the relative contributions of prenatal environmental factors and genotype on DNA methylation in neonatal blood at variably methylated regions (VMRs) in 4 independent cohorts (overall *n* = 2365). We use Akaike’s information criterion to test which factors best explain variability of methylation in the cohort-specific VMRs: several prenatal environmental factors (E), genotypes in cis (G), or their additive (G + E) or interaction (GxE) effects. Genetic and environmental factors in combination best explain DNAm at the majority of VMRs. The CpGs best explained by either G, G + E or GxE are functionally distinct. The enrichment of genetic variants from GxE models in GWAS for complex disorders supports their importance for disease risk.

## Abstract (web summary)

Environmental influences during prenatal development may have implications for health and disease later in life. Here, Czamara et al. assess DNA methylation in cord blood from new-born under various models including environmental and genetic effects individually and their additive or interaction effects.

## Introduction

Foetal or prenatal programming describes the process by which environmental events during pregnancy influence the development of the embryo with on-going implications for future health and disease. Several studies have shown that the in utero environment is associated with disease risk, including coronary heart disease^1,2^, type 2 diabetes^3^, childhood obesity^4,5^ as well as psychiatric problems^6^ and disorders^7–9^.

Environmental effects on the epigenome, for example, via DNA methylation, could lead to sustained changes in gene transcription and thus provide a molecular mechanism for the enduring influences of the early environment on later health^10^. Smoking during pregnancy influences widespread and highly reproducible differences in DNA methylation at birth^11^. Less dramatic effects have been reported for maternal body mass index (BMI)^12^, pre-eclampsia and gestational diabetes^13,14^. Possible epigenetic changes as a consequence of prenatal stress are less well established^15^. Some of these early differences in DNA methylation persist, although attenuated, through childhood^11,16^ and might be related to later symptoms and indicators of disease risk, including BMI during childhood^17,18^ or substance use in adolescence^19^. These data emphasise the potential importance of the prenatal environment for the establishment of inter-individual variation in the methylome as a predictor or even mediator of disease risk trajectories.

In addition to the environment, the genome plays an important role in the regulation of DNA methylation. To this end, the impact of genetic variation, especially of single nucleotide polymorphisms (SNPs) on DNA methylation in different tissues, has resulted in the discovery of a large number of methylation quantitative trait loci (meQTLs, i.e., SNPs significantly associated with DNA methylation status^20^). These variants are primarily in cis, i.e., at most 1 million base pairs away from the DNA methylation site^20–22^ and often co-occur with expression QTLs or other regulatory QTLs^23–25^. The association of meQTLs with DNA methylation is relatively stable throughout the life course^21^. In addition, SNPs within meQTLs are strongly enriched for genetic variants associated with common disease in large genome-wide association studies (GWAS) such as BMI, inflammatory bowel disease, type 2 diabetes or major depressive disorder^21,23,24,26^.

Environmental and genetic factors may act in an additive or multiplicative manner to shape the epigenome to modulate phenotype presentation and disease risk^27^. However, very few studies have so far investigated the joint effects of environment and genotype on DNA methylation, especially in a genome-wide context. Klengel et al.^28^, for instance, reported an interaction of the FK506 binding protein 5 gene (*FKBP5)* SNP genotype and childhood trauma on *FKBP5* methylation levels in peripheral blood cells, with trauma associated changes only observed in carriers of the rare allele. The most comprehensive study of integrated genetic and environmental contributions to DNA methylation so far was performed by Teh et al.^29^. This study examined variably methylated regions (VMRs), defined as regions of consecutive CpG-sites showing the highest variability across all methylation sites assessed on the Illumina Infinium HumanMethylation450 BeadChip array. In a study of 237 neonate methylomes derived from umbilical cord tissue, the authors explored the proportions of the influence of genotype vs. prenatal environmental factors such as maternal BMI, maternal glucose tolerance and maternal smoking on DNA methylation at VMRs. They found that 75% of the VMRs were best explained by the interaction between genotype and environmental factors (GxE) whereas 25% were best explained by SNP genotype and none by environmental factors alone. Collectively, these studies highlight the importance of investigating the combination of environmental and genetic contributions to DNA methylation and not only their individual contribution.

The main objective of the present study is to extend our knowledge of combined effects of prenatal environment and genetic factors on DNA methylation at VMRs. Specifically, this is addressed by: (1) assessing the stability of the best explanatory factors across different cohorts and whether this extends to all environmental factors, (2) dissecting differences between additive and interactive effects of gene and environment not explored in Teh et al., (3) testing whether VMRs influenced by genetic and/or environmental factors might have a different predicted impact on gene regulation and (4) evaluating the relevance of genetic variants that interact with the environment to shape the methylome for their contribution to genetic disease risk.

Our results show that across cohorts genetic variants in combination with prenatal environment are the best predictors of variance in DNA methylation. We observe functional differences of both the genetic variants and the methylation sites best explained by genetic or additive and interactive effects of genes and environment. Finally, the enrichment of genetic variants within additive as well as interactive models in GWAS for complex disorders supports the importance of these environmentally modified methylation quantitative trait loci for disease risk.

## Results

### Cohorts and analysis plan

**Fig. 1:**
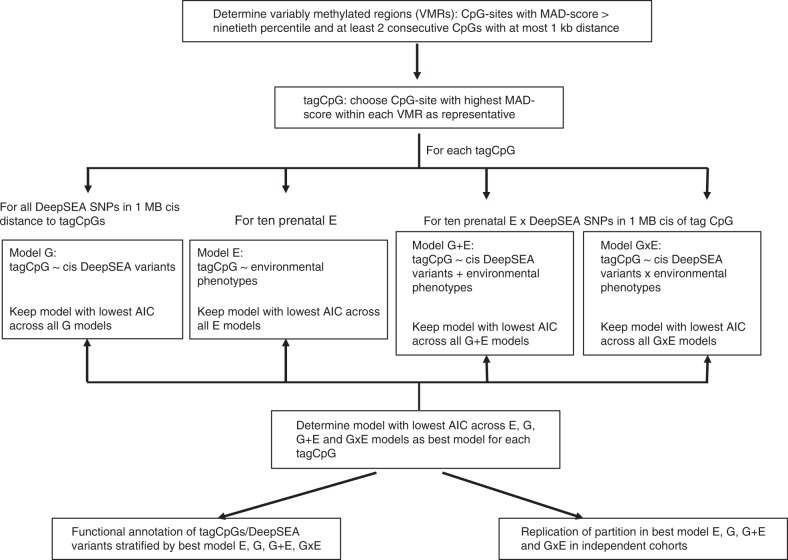
Flow diagram of VMR analysis

We investigated the influence of the prenatal environment and genotype on VMRs in the DNA of 2365 newborns within 4 different cohorts: Prediction and Prevention of Pre-eclampsia and Intrauterine Growth Restrictions (PREDO, cordblood)^30^, the UCI cohort (refs. ^31–33^, heel prick), the Drakenstein Child Health Study (DCHS, cordblood)^34,35^ and the Norwegian Mother and Child Cohort Study (MoBa, cordblood^36^). A description of the workflow of this manuscript is given in Fig. 1 and the details for each of the cohorts are given in Table 1.

**Table 1:** Overview of investigated cohorts

Cohort	PREDO I	PREDO II	DCHS I	DCHS II	UCI	MoBa
Sample size	817	146	107	151	121	1023
Methylation array	Illumina 450 K	Illumina EPIC	Illumina 450 K	Illumina EPIC	Illumina EPIC	Illumina 450 K
Methylation data processing	Funnorm and Combat	Funnorm and Combat	SWAN and Combat	BMIQ and Combat	Funnorm and Combat	BMIQ and Combat
SNP genotyping	Illumina Human Omni Express Exome	Illumina Human Omni Express Exome	Illumina PsychArray	Illumina GSA	Illumina Human Omni Express	Illumina HumanExome Core
Infant gender male	433 (53.0%)	75 (51.4%)	63 (58.8%)	83 (55.0%)	65 (53.7%)	478 (46.7%)
Maternal age mean (sd)	33.28 (5.79)	32.25 (4.92)	26.27 (5.87)	27.42 (5.93)	28.47 (4.91)	29.92 (4.32)
Partity mean (sd)	1.05 (1.02)	0.87 (1.03)	0.98 (1.12)	1.09 (1.07)	1.11 (1.15)	0.83 (0.88)
Caesarian section	169 (20.7%)	36 (24.7%)	19 (17.6%)	35 (23.2%)	37 (30.6%)	228 (22.3%)
Pre-pregnancy BMI mean (sd)	27.42 (6.40)	25.37 (5.79)	Not available	Not available	27.90 (6.44)	24.05 (4.19)
Maternal smoking yes	Exclusion criterion	Exclusion criterion	7.40 (10.52)^a^	4.94 (9.43)^a^	10 (8.2%)	148 (14.4%)
Gestational diabetes yes	183 (22.4%)	19 (13.0%)	No cases available	No cases available	9 (7.4%)	15 (1.5%)
Hypertension yes	275 (33.7%)	31 (21.2%)	2 (0.19%)	2 (1.3%)	7 (5.8%)	50 (4.9%)
Betamethasone treatment yes	35 (4.3%)	2 (1.5%)	Not available	Not available	No cases available	Not available
Anxiety score mean (sd)	33.93 (7.90)^b^	34.43 (8.38)^b^	5.70 (4.15)^c^	5.32 (3.91)^c^	1.67 (0.41)^d^	4.79 (1.36)^e^
Depression score mean (sd)	11.34 (6.47)^f^	11.53 (6.98)^f^	17.64 (12.10)^g^	12.52 (11.55)^g^	0.68 (0.41)^h^	5.24 (1.57)^e^

^a^ Based on ASSIST Tobacco Score

^b^ STAI sum scores

^c^ SRQ-20

^d^ STAI average scores

^e^ Based on Hopkins Symptom Checklist

^f^ CESD sum scores

^g^ BDI-II

^h^ CESD average score

We analysed 963 cord blood samples from the PREDO cohort with available genome-wide DNA methylation and genotype data. Of these samples, 817 had data on the Illumina 450k array (PREDO I) and 146 on the Illumina EPIC array (PREDO II). The main analyses are reported for PREDO I, and replication and extension of the results is shown for PREDO II as well as for three independent cohorts including 121 heel prick samples (UCI cohort, EPIC array) as well as 258 (DCHS, 450 K and EPIC array) and 1023 cord blood samples (MoBa, 450 K array). We tested 10 different prenatal environmental factors covering a broad spectrum of prenatal phenotypes (see Table 1) (referred to as E), as well as cis SNP genotype (referred to as G), i.e., SNPs located in at most 1MB distance to the specific CpG, additive effects of cis SNP genotype and prenatal environment (G + E) and cis SNP×environment interactions (GxE) for association with DNA methylation levels (see Fig. 1). We then assessed for each VMR independently which model described the variance of DNAm best using Akaike’s information criterion (AIC)^37^. In all models, we corrected for child’s gender, ethnicity (using MDS-components), gestational age as well as estimated cell proportions to account for cellular heterogeneity.

### Variably methylated regions

We first identified candidate VMRs, defined as regions of CpG-sites showing the highest variability across all methylation sites. In PREDO I, we identified 10,452 variable CpGs that clustered into 3982 VMRs (see Supplementary Data 1). Most VMRs (*n* = 2683) include 2 CpGs. As detailed in Supplementary Note 1, the distribution of methylation levels of CpGs within these VMRs is unimodal, (see Supplementary Fig. 1A), VMRs are enriched in specific functional regions of the genome, correlate with differences in gene expression, and overlap with sites associated with specific prenatal environmental factors.

To examine the factors that best explain the variance in methylation in these functionally relevant sites, we chose the CpG-site with the highest MAD score as representative of the VMR. These CpGs are named tagCpGs. The correlation between methylation levels of tagCpG and average methylation of the respective VMR was high (mean *r* = 0.85, sd *r* = 0.08), suggesting that the tag CpGs are valid representatives of their VMRs. Furthermore, tagCpGs are mainly uncorrelated with each other (mean *r* = 0.03, sd = 0.12).

### Which models explain methylation of tagCpGs best?

**Fig. 2:**
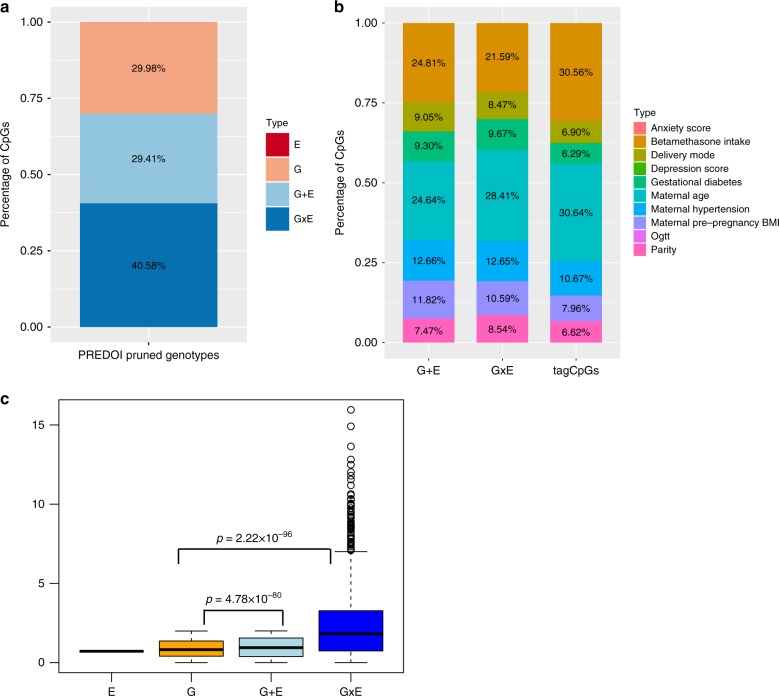
VMR analysis in pruned PREDO I dataset. **a** Percentage of models (G, E, GxE or G + E) with the lowest AIC explaining variable DNA methylation using the PREDO I dataset with pruned SNPs. **b** Distribution of the different types of prenatal environment included in the E model with the lowest AIC (right), in the combinations yielding the best model GxE (middle), or the best model G + E models (left). To increase readability all counts <3% have been omitted. **c** DeltaAIC, i.e, the difference in AIC, between best model and next best model, stratified by the best model. *Y*-axis denotes the delta AIC and the *X*-axis the different models. The median is depicted by a black line, the rectangle spans the first quartile to the third quartile, whiskers above and below the box show the location of minimum and maximum beta-values. *P*-values are based on Wilcoxon-tests

We next compared the fit of four models for each of the 3,982 tagCpGs (see Fig. 1): best SNP (G model), best environment (E model), SNP+ environment (G + E model) and SNP× environment (GxE model). Association results for each model are listed in Supplementary Data 2–5. For each tagCpG, the model with the lowest AIC was chosen as the best model (see Methods section). In total, 40.6% of tagCpGs were best explained by GxE (*n* = 1616), followed by G (30%, *n* = 1, 194) and G + E (29%, *n* = 1171) (Fig. 2a). E explained most variance in one tagCpG. All tag CpGs and the respective SNPs and environments from the best model are listed in Supplementary Data 6–8 and Supplementary Table 1.

With regard to environmental factors, 27.0% of tagCpGs best explained by the G + E model were associated with environmental factors related with stress or, in particular, glucocorticoids (i.e., maternal betamethasone treatment), 40.8% with general maternal factors (mostly maternal age) and 32.20 % with factors related to metabolism (pre-pregnancy BMI, hypertension, gestational diabetes). For best model GxE tagCpGs, the proportions of environmental factors were similar with 22.2, 44.1 and 33.7%, respectively (see Fig. 2b).

We next looked into the delta AIC, i.e., the difference between the AIC of the best model to the AIC of the next best model (see Supplementary Note 2). GxE models appear to be winning by a significantly larger AIC margin over the next best model, when compared to the other types of winning models (see Fig. 2c).

### DeepSEA prediction of SNP function

We were next interested in understanding the functionality of both the VMRs as well as the associated SNPs in the G, GxE and G + E models. For this we restricted the analyses only to potentially functional relevant SNPs using DeepSEA^38^ and not all linkage disequilibrium (LD)-pruned SNPs as described above. DeepSEA, a deep neural network pretrained with DNase-seq and ChIP-seq data from the ENCODE^39^ project, predicts the presence of histone marks, DNase hypersensitive regions (DHS) or TF binding for a given 1 kb sequence. The likelihood that a specific genetic variant influences regulatory chromatin features is estimated by comparing predicted probabilities of two sequences where the bases at the central position are the reference and alternative alleles of a given variant. We reran the four models now restricting the cis-SNPs to those 36,241 predicted DeepSEA variants that were available in our imputed, quality-controlled genotype dataset.

Top results for models including G, GxE and G + E are depicted in Supplementary Data 9–12.

**Fig. 3:**
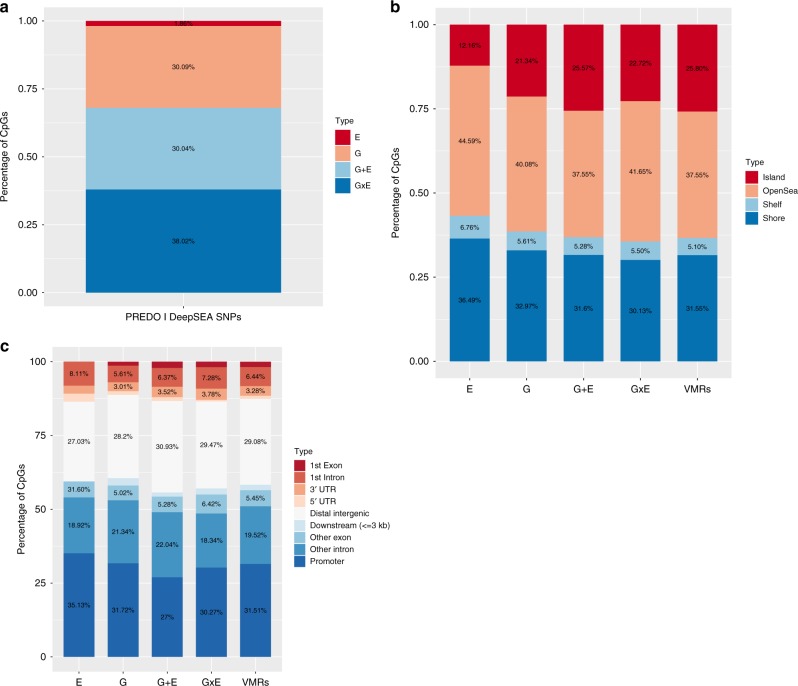
VMR analysis in DeepSEA annotated SNPs in PREDO I dataset. **a** Percentage of models (G, E, GxE or G + E) with the lowest AIC explaining variable DNA methylation using the PREDO I dataset with DeepSEA annotated SNPs. **b** Distribution of the locations of all VMRs and tagVMRs with best model E, G, G + E and GxE on the 450k array using only DeepSEA variants in relationship to CpG-Islands based on the Illumina 450 K annotation. **c** Distribution of gene-centric locations of all VMRs and tagVMRs with best model E, G, G + E and GxE on the 450k array using only DeepSEA variants

Results were comparable to what we observed before: 1195 (30.09%) of tagCpGs presented with best model G, 1193 CpGs (30.04%) with best model G + E, 1510 CpGs (38.02%) with best model GxE and 74 CpGs (1.86%) with best model E (Fig. 3a) and also showed similar differences in delta-AIC and proportions of E categories (see Supplementary Note 3). Only 10 tagCpGs did not present with any DeepSEA variant within 1MB distance in cis and were therefore not further considered. All respective CpG-environment-DeepSea SNP combinations are depicted in Supplementary Data 13–16.

The distribution of best models was not influenced by the degree of variability of DNA methylation, but was comparable across the whole range of DNA methylation variation (see Supplementary Note 4 and Supplementary Fig. 2). A slight enrichment for G + E models was observed in longer VMRs with at least 3 CpGs (*p* = 9.00 × 10^−06^, OR = 1.39, Fisher-test, see Supplementary Fig. 3).

In conclusion, also when we focus on potentially functionally relevant SNPs, it is the combination of genotype and environment which best explains VMRs.

We observed that, as expected, different types of exposures or maternal factors have different relative impact on DNA methylation (see Supplementary Note 5). However, even for those exposures with the highest fraction of VMRs best explained by E alone, combined models of G + E and GxE remain the best models in even higher fractions of VMRs (see Supplementary Fig. 4B).

### Functional annotation of different best models

Focusing on combinations between tagCpGs, environmental factors and DeepSEA variants, we found functional differences for both the SNPs as well as the tagCpGs (see Supplementary Note 6) within the different models. Overall, 895 DeepSEA variants were uniquely involved in best G models, 905 were uniquely in best G + E models and 1162 uniquely in best GxE models. As a DeepSEA variant can be in multiple 1 MB-cis windows around the tagCpGs, several DeepSEA variants were involved in multiple best models: 138 DeepSEA variants overlapped between G and GxE, 118 between G and G + E and 147 between GxE and G + E VMRs. We observed no significant differences with regard to gene-centric location for DeepSEA variants involved only in G models, only in G + E models or in multiple models. However, DeepSEA variants involved only in GxE models were significantly depleted for promoter locations (*p* = 3.92 × 10^−02^, OR = 0.79, Fisher-test, see Supplementary Fig. 5A).

Although no significant differences were present, DeepSEA SNPs involved in the G and G + E model were located in closer proximity to the specific CpG (model G: mean absolute distance = 256.8 kb, sd = 291.2 kb, model G + E: mean absolute distance = 244.8 kb, sd = 284.0 kb, Supplementary Fig. 5B) whereas DeepSEA SNPs involved in GxE models (mean absolute distance = 352.6 kb, sd = 305.3 kb) showed broader peaks around the CpGs.

**Fig. 4:**
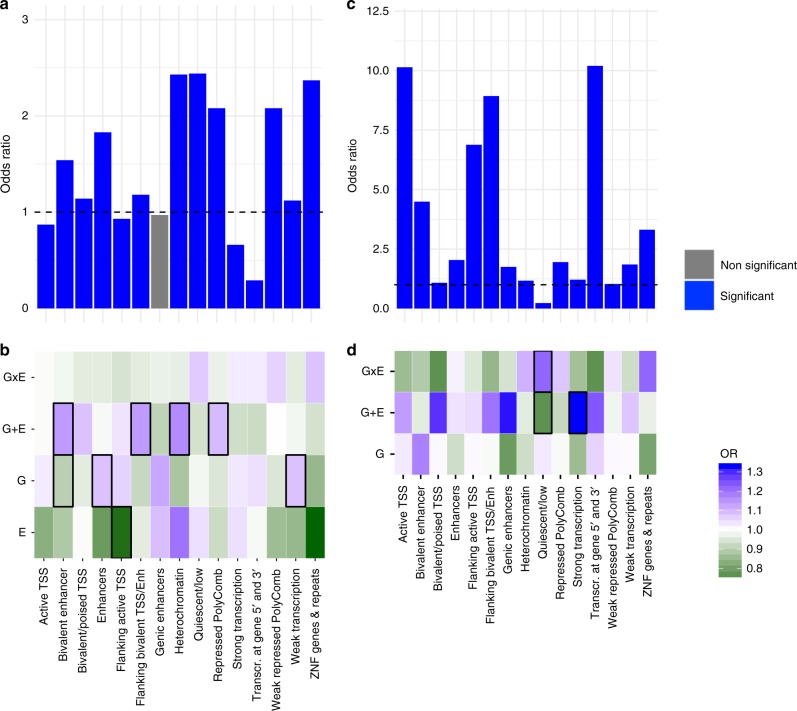
Functional annotation of VMR-mapping in DeepSEA annotated SNPs in PREDO I dataset. **a** Histone mark enrichment for all VMRs. The Y-axis denotes the fold enrichment/depletion as compared to no-VMRs. Blue bars indicate significant enrichment/depletion, grey bars non-significant differences based on Fisher-tests. **b** Histone mark enrichment for tagVMRs with best model E, G, G + E and GxE relative to all VMRs. Green colour indicates depletion, red colour indicates enrichment. Thick black lines around the rectangles indicate significant enrichment/depletion based on Fisher-tests. **c** Histone mark enrichment for all DeepSEA variants in the dataset. Blue bars indicate significant enrichment/depletion based on Fisher-tests. **d** Histone mark enrichment for all DeepSEA variants involved in models where either G, G + E or GxE is the best model as compared to all tested DeepSEA variants. Green colour indicates depletion, red colour indicates enrichment. Thick black lines around the rectangles indicate significant enrichment/depletion based on Fisher-tests

With regards to histone marks, DeepSEA variants in general were enriched across multiple histone marks indicative of active transcriptional regulation (Fig. 4c). DeepSEA variants involved in best model G + E showed further enrichment for strong transcription (*p* = 7.19 × 10^−03^, OR = 1.34, Fisher-test) as well as depletion for quiescent loci (*p* = 7.17 × 10^−03^, OR = 0.78, Fisher-test). In contrast, GxE DeepSEA variants were significantly enriched in these regions (*p* = 2.62 × 10^−02^, OR = 1.22, Fisher-test, Fig. 4d).

Taken together, these analyses indicate that both the genetic variants and the VMRs in the different best models (G, GxE and G + E) preferentially annotate to functionally distinct genomics regions.

### Replication of best models in independent cohorts

To assess whether the relative distribution of the best models for VMRs and DeepSEA variants was stable across different samples, we assessed the relative distribution of these models in 3 additional samples (DCHS I and DCHS II, UCI and PREDOII) with VMR data both from the Illumina 450 K as well as the IlluminaHumanEPIC arrays. Information on these cohorts is summarised in Table 1 and the number of VMRs, the distribution of VMR methylation levels, VMR length and specific SNP information are given in Supplementary Note 7 and Supplementary Fig. 6.

**Fig. 5:**
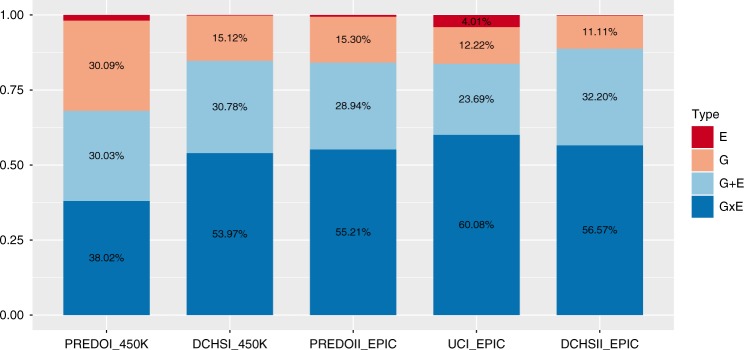
VMR analysis in PREDO I and replication datasets. Percentage of models (G, E, GxE or G + E) with the lowest AIC explaining variable DNA methylation in PREDO I (450 K), DCHS I (450 K), PREDO II (EPIC), UCI (EPIC) and DCHS II (EPIC)

While major maternal factors overlapped among the cohorts - such as maternal age, delivery method, parity and depression during pregnancy - there were also differences, as the non-PREDO cohorts did not include betamethasone treatment but additionally included maternal smoking (see Table 1). Despites these differences and differences in the total number of VMRs, the overall pattern remained stable: in all 4 analyses, DCHS I, DCHS II, UCI and PREDO II, we replicated that E alone models almost never explained most of the variances, while G alone models explained the most variance in up to 15% of the VMRs; G + E in up to 32%; and GxE models in up to 60% (see Fig. 5 and Table 2).

**Table 2:** VMRs and best models across cohorts

Cohort	PREDO I	PREDO II	DCHS I	DCHS II	UCI
Sample-size	817	146	107	151	121
Methylation array	Illumina 450 K	Illumina EPIC	Illumina 450 K	Illumina EPIC	Illumina EPIC
# VMRs	3972	8547	6072	10,005	9525
Proportion: best model E (%)	2.0	<1	<1	<1	4.1
Best model G (%)	30.0	15.0	15.8	11.5	12.8
Best model G + E (%)	30.0	29.0	29.8	32.1	24.1
Best model GxE (%)	38.0	56.0	54.3	56.3	59.0

The importance of including G for a best model fit could also be observed for maternal smoking, described as one of the most highly replicated factors shaping the newborns’ methylome^11^ and present in the replication but not the discovery cohort PREDO I. These analyses are detailed in Supplementary Note 8.

We were also able to replicate our finding showing that GxE VMRs were enriched for OpenSea positions with a trend on the 450 K array (DCHS I, OR = 1.11, *p* = 5.03 × 10^−02^, Fisher-test) and significantly for the EPIC array data (PREDOII: *p* = 2.96 × 10^−06^, OR = 1.29, UCI: *p* = 3.79 × 10^−02^, OR = 1.09, DCHSII: *p* = 2.91 × 10^−04^, OR = 1.16, Fisher-tests). For all additional cohorts, the delta AIC for best model GxE to the next best model was also significantly higher as compared to CpGs with G, E or G + E as the best model.

**Fig. 6:**
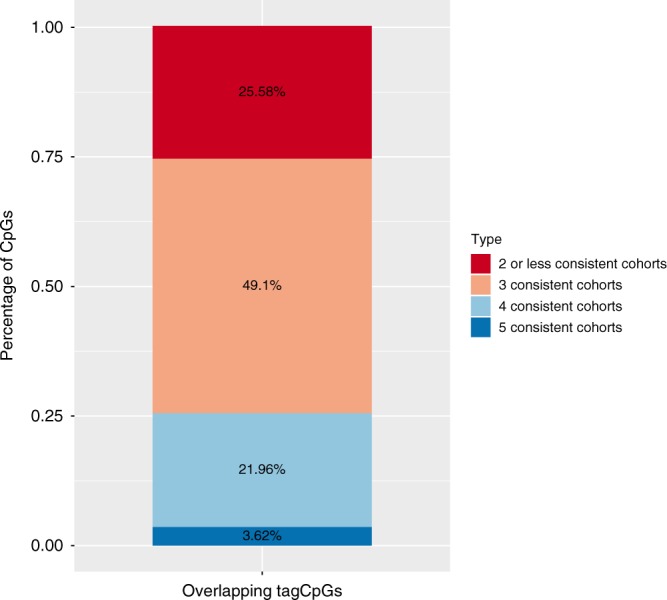
Consistency of best models across cohorts. Percentage of consistent best models in overlapping tag CpGs of PREDO I (450 K), DCHS I (450 K), PREDO II (EPIC), UCI (EPIC) and DCHS II (EPIC). Overlapping VMRs included significantly more CpGs as compared to all VMRs (*p* < 2.2 × 10^−16^, Wilcoxon-test, mean = 4.43)

Overall, 387 tag CpGs overlapped between PREDO I, PREDO II, DCHS I and DCHS II (see Supplementary Fig. 7), which allowed us to test the consistency of the best models for specific VMRs across the different cohorts. Over 70% of the overlapping tagCPGs showed consistent best models in at least 3 cohorts (see Fig. 6) with GxE being the most consistent model (for over 60% of consistent models, see Supplementary Fig. 8). Focusing only on EPIC data (PREDO II, DCHSII and UCI), we identified more, namely 2091, tag CpGs that overlap across the three cohorts and here 86% show a consistent best model in at least two of the three cohorts, despite differences in study design, prenatal phenotypes and ethnicity.

Thus, the additional cohorts not only showed a consistent replication of the proportion of the models best explaining variance of VMRs but also consistency of the best model for specific VMRs. Within this context, we observed the GxE models were the most consistent models across the cohorts (see Supplementary Fig. 8), with 85% of the CpGs with consistent models across 5 cohorts having GxE as the best model. Furthermore, we could validate specific GxE combinations between PREDO I and MoBa as shown as in the Supplementary Note 9, in Supplementary Data 17 and 18 and in Supplementary Fig. 9.

### Disease relevance

**Fig. 7:**
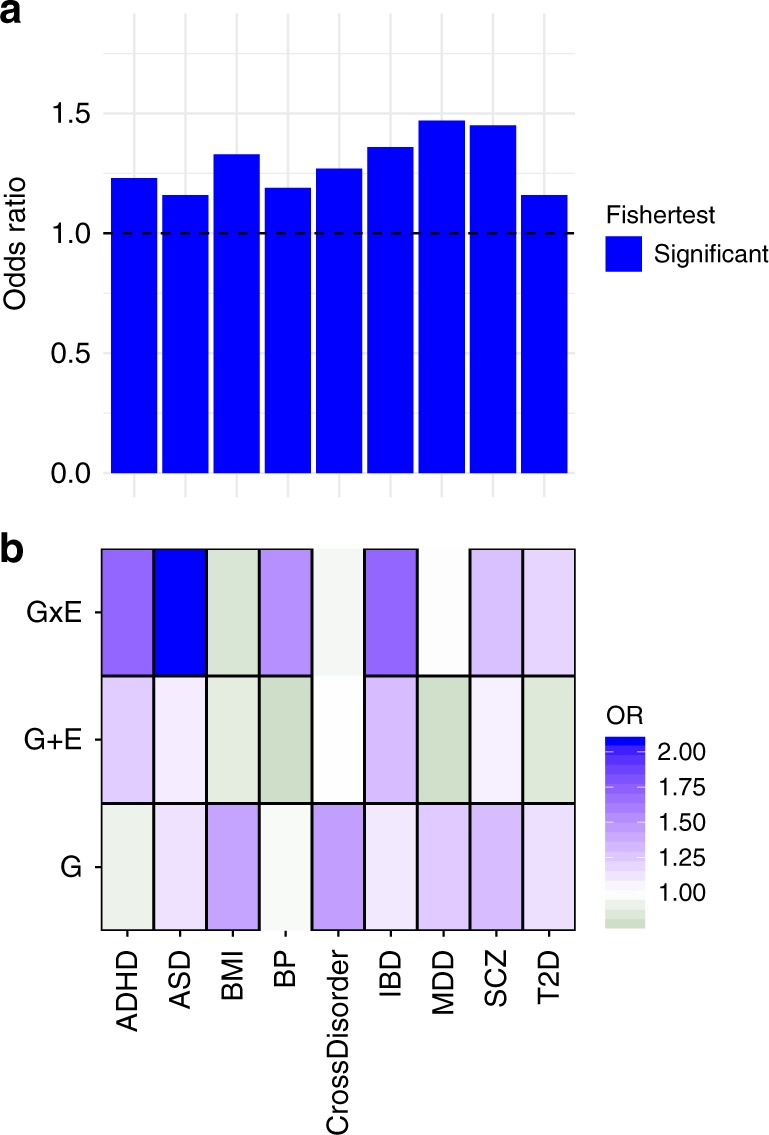
Enrichment of DeepSEA variants for GWAS associations. **a** Enrichment for nominal significant GWAS associations for all tested DeepSEA variants and their LD proxies for GWAS for ADHD (attention-deficit hyperactivity disorder), ASD (autism spectrum disorder), BMI (body mass index), BP (bipolar disorder), CrossDisorder, IBD (inflammatory bowel disease), MDD (major depressive disorder), SCZ (schizophrenia) and T2D (Type 2 diabetes). The *Y*-axis denotes the fold enrichment with regard to non-DeepSEAvariants. Blue bars indicate significant enrichment/depletion based on Fisher-tests. **b** Enrichment for nominal significant GWAS hits for DeepSEA variants and their LD proxies involved in best models with G, G + E or GxE as compared to all tested DeepSEA variants. Green colour indicates depletion, red colour indicates enrichment. Thick black lines around the rectangles indicate significant enrichment/depletion based on Fisher-tests

Finally, we tested whether functional DeepSEA SNPs involved in only G, only GxE and only G + E models in PREDO I for their enrichment in GWAS hits. We used all functional SNPs and their LD proxies (defined as *r*^2^ of at least 0.8 in the PREDO cohort and in maximal distance of 1MB to the target SNP) and performed enrichment analysis with the overlap of nominal significant GWAS hits. We selected for a broad spectrum of GWAS, including GWAS for complex disorders for which differences in prenatal environment are established as risk factors, but also including GWAS on other complex diseases. For psychiatric disorders, we used summary statistics of the Psychiatric Genomics Consortium (PGC) including association studies for autism^40^, attention-deficit-hyperactivity disorder^41^, bipolar disorder^42^, major depressive disorder^43^, schizophrenia^44^ and the cross-disorder associations including all five of these disorders^45^. Additionally, we included GWAS of inflammatory bowel disease^46^, type 2 diabetes^47^ and for BMI^48^. Nominal significant GWAS findings were enriched for DeepSEA variants and their LD proxies per se across psychiatric as well as non-psychiatric diseases (Fig. 7a). However, G, GxE and G + E DeepSEA variants showed a differential enrichment pattern above all DeepSEA variants (Fig. 7b), with strongest enrichments of GxE DeepSEA variants in GWAS of autism spectrum disorder (*p* < 2.20 × 10^−16^, OR = 2.07 above DeepSEA, Fisher-test), attention-deficit-hyperactivity disorder (*p* < 2.20 × 10^−16^, OR = 1.71, Fisher-test) and inflammatory bowel disease (*p* < 2.20 × 10^−16^, OR = 1.71, Fisher-test) and G + E DeepSEA variants in GWAS for attention-deficit-hyperactivity disorder (*p* = 9.54 × 10^−36^, OR = 1.23, Fisher-test) and inflammatory bowel disease (*p* = 1.85 × 10^−52^, OR = 1.30, Fisher-test). While SNPs with strong main meQTL effects such as those within G and G + E models have been reported to be enriched in GWAS for common disease, we now also show this for SNPs within GxE models that often have non-significant main G effects.

## Discussion

We evaluated the effects of prenatal environmental factors and genotype on DNA methylation at VMRs identified in neonatal blood samples. We found that most variable methylation sites were best explained by either genotype and prenatal environment interactions (GxE) or additive effects (G + E) of these factors, followed by main genotype effects. This pattern was replicated in independent cohorts and underscores the need to consider genotype in the study of environmental effects on DNA methylation.

In fact, VMRs best explained by G, G + E or GxE and their associated functional genetic variants were located in distinct genomic regions, suggesting that different combinatorial effects of G and E may impact VMRs with distinct downstream regulatory effects and thus possibly context-dependent impact on cellular function. We also observed that functional variants with best models G, G + E or GxE, all showed significant enrichment within GWAS signals for complex disorders beyond the enrichment of the functional variants themselves. While this was expected for G and G + E models based on results from previous studies^21,23,24,26^, it was surprising for GxE SNPs, as these often do not have highly significant main genetic effects. Their specific enrichment in GWAS for common disorders supports the importance of these genetic variants that moderate environmental impact both at the level of DNA methylation but also, potentially, for disease risk.

The fact that GxE and G + E best explained the majority of VMRs (see Fig. 5) and that GxE models were selected by a larger margin than the other models (see Fig. 2c) was consistently found across all tested cohorts. These findings are in line with a previous report by Teh et al.^29^ who performed a similar analysis based on AIC in umbilical cord tissue. Differences to the findings by Teh et al. are discussed in the Supplemental Discussion. Using data from four different cohorts, we not only saw comparable proportions of VMRs best explained by the different models, but also saw in the VMRs common across cohorts that specific VMRs had consistent best models (see Fig. 6). This is in line with the fact that VMRs best explained by G, GxE or G + E show functional differences and may differentially impact gene regulation.

In addition to consistent findings using AIC-based approaches, we also observed some indication for validation of individual GxE and G + E combinations on selected VMRs using p-value based criteria, with a small number of specific G + E and GxE effects on VMRs replicating between the PREDO I and the MoBa cohort. The low number of specific replications could be due to lack of overall power as well as larger differences in prenatal factors between these two cohorts (see Table 1). As shown in Supplementary Fig. 4B, which specific G and E combinations best explain VMRs is also dependent on the specific prenatal factors. Larger and more homogenous cohorts regarding exposures will be needed for such analyses to be more conclusive.

While E alone was rarely the best model, it should be pointed out that main environmental effects on DNA methylation were observed (see Supplementary Data 3), and consistent with previous large meta-analyses such as in the case of maternal smoking (see Supplementary Note 7). Within the MoBa cohort, the cohort with the largest proportion of maternal smoking, 10% of all tagCpGs were best explained by maternal smoking alone. However, in all other cohorts, where smoking was less prevalent, the inclusion of genotypic effects in addition to maternal smoking explained more of the variance. This supports that while main E effects on the newborn methylome are present, genotype is an important factor that, in combination with E, may explain even more of the variance in DNA methylation.

VMRs best explained by either E, G, G + E or GxE and their associated functional SNPs were enriched for distinct genomics locations and chromatin states (see Fig. 4), suggesting that VMRs moderated by different combinations of G and E may in fact have distinct functional roles in gene regulation. Overall, VMRs best explained by GxE were consistently enriched for regions annotated to the OpenSea regions with lower CpG density and located farthest from CpG Islands^49^. Open Sea regions have been reported to be enriched for environmentally-associated CpGs with for example exposure to childhood trauma^50^ and may harbour more long-range enhancers.

In addition to their position relative to CpG islands and their CpG content, G, GxE and G + E VMRs and their associated functional SNPs also showed distinct enrichments for chromatin marks. Compared to 450 K VMRs in general, VMRs with GxE as the best models were relatively depleted in regions surrounding the TSS, while VMRs with G + E were relatively enriched in these regions (see Fig. 4), suggesting that GxE VMRs are located at more distance from the TSS than G + E VMRs. To better map the potential functional variants in these models and to compare methylation-associated SNPs from a regulatory perspective, we used DeepSEA^38^, a machine learning algorithm that predicts SNP functionality from the sequence context based on sequencing data for different regulatory elements in different cell lines using ENCODE data^39^. We identified the SNPs with putatively functional consequences on regulatory marks by DeepSEA and compared putative regulatory effects of G, G + E and GxE hits. Relative to the imputed non-DeepSEA SNPs contained in our dataset, these predicted functional DeepSEA SNPs were enriched for TSS and enhancer regions and depleted for quiescent regions, supporting their relevance in regulatory processes (see Fig. 4). Compared to DeepSEA SNPs overall, DeepSEA SNPs within the three different best models also showed distinct enrichment or depletion patterns. Similar to GxE VMRs, likely functional GxE SNPs also showed a relative depletion in TSS regions while G + E SNPs showed enrichment in genic enhancers. Overall, both the VMRs as well as the associated functional SNPs appear to be in distinct regulatory regions, depending on their best model. In addition, GxE functional SNP and tagCpGs were located farther apart than SNP/tagCpG pairs within G or G + E models (see Supplementary Fig. 5B), supporting a more long-range type of regulation in GxE interactions on molecular traits as compared to all genes; a similar relationship has been reported previously for GxE with regard to gene expression in *C. elegans*^51,52^.

SNPs associated with differences in gene expression but also DNA methylation have consistently been shown to be enriched among SNPs associated with common disorders in GWAS^21,24,26,53^. The functional genetic variants that were within G, GxE or G + E models predicting variable DNA methylation were even enriched in GWAS association results (beyond the baseline enrichment of DeepSea SNPs per se). The fact that such enrichment was observed for not only G and G + E SNPs, with strong main genetic effects, but also for GxE SNPs, with smaller to sometimes no main genetic effect on DNA methylation underscores the importance of also including SNPs within GxE models in the functional annotation of GWAS. A detailed catalogue of meQTLs that are responsive to environmental factors could support a better pathophysiological understanding of diseases for which risk is shaped by a combination of environment and genetic factors.

Finally, we want to note the limitations of this study. First, we restricted our analyses to specific DNA methylation array contents that are inherently biased as compared to genome-wide bisulfite sequencing, for example. In addition, we restricted our analysis to VMRs, which also limits the generalisability of the findings, but also has advantages. Ong and Holbrooke^54^ showed that this approach increases statistical power. Furthermore, VMRs are enriched for enhancers and transcription factor binding sites, overlap with GWAS hits^55^ and are associated with gene expression of nearby genes at these sites^56^. VMRs in this study presented with intermediate methylation levels which have been shown to be enriched in regions of regulatory function, like enhancers, exons and DNase I hypersensitivity sites^57^. Hence, the effects of genotypes on DNA methylation levels in VMRs might be higher as compared to less variable CpG-sites. In addition, genotypes are measured with much less error as compared to environmental factors which may also reduce the overall explained variance in large cohorts.

Second, it has been reported that different cell types display different patterns of DNA methylation^55^. Therefore, the most variable CpG-sites may also include those that reflect differences in cord blood cell type proportions. To address this issue, all analyses were corrected for estimated cell proportions to the best of our current availability, so that differences in cell type proportion likely do not account for all of the observed effects. However, only replication in specific cell types will be able to truly assess the proportion of VMRs influenced by this.

Third, we used the AIC as main criterion for model fit^37^ which is equivalent to a penalised likelihood-function. There are a variety of other model selection criteria^58^ and choosing between these is an ongoing debate which also depends on the underlying research question. We decided to use the AIC as one of our main aims was to compare our results with the study of Teh et al.^29^ in which this criterion was applied and as this method maybe more powerful for detecting GxE than for example model selection criteria based on lowest *p*-values.

Fourth, all reported interactions are statistical interactions and limited to a *cis* window around the CpG-site. Further experiments are required to assess whether these would also reflect biological/mechanistic interactions. Much larger cohorts will be needed to assess potential *trans* effects. Additional inclusion of further covariates such as maternal smoking or maternal age may further modify the effects of specific Es but is beyond the scope of this manuscript.

Fifth, as summarised in Table 1, results presented are based on cohorts which differ in ethnicity, assessed phenotypes, methylation and SNP arrays, processing pipelines and sample sizes. While all these factors may contribute to differences in the proportions of models across the cohorts, it also suggests that our findings are quite robust to these methodological issues.

Finally, our analyses are restricted to DNA methylation in neonatal blood and to pregnancy environments. Whether similar conclusions can be drawn for methylation levels assessed at a later developmental stage needs to be investigated.

We tested whether genotype, a combination of different prenatal environmental factors and the additive or the multiplicative interactive effects of both mainly influence VMRs in the newborn’s epigenome. Our results show that G in combination with E are the best predictors of variance in DNA methylation. This highlights the importance of including both individual genetic differences as well as environmental phenotypes into epigenetic studies and also the importance of improving our ability to identify environmental associations. Our data also support the disease relevance of variants predicting DNA methylation together with the environment beyond main meQTL effects, and the view that there are functional differences of additive and interactive effects of genes and environment on DNA methylation. Improved understanding of these functional differences may also yield novel insights into pathophysiological mechanisms of common non-communicable diseases, as risk for all of these disorders is driven by both genetic and environmental factors.

## Methods

### The PREDO cohort

The Prediction and Prevention of Preeclampsia and Intrauterine Growth Restriction (PREDO) Study is a longitudinal multicenter pregnancy cohort study of Finnish women and their singleton children born alive between 2006 and 2010 ^30^. We recruited 1,079 pregnant women, of whom 969 had one or more and 110 had none of the known clinical risk factors for preeclampsia and intrauterine growth restriction. The recruitment took place when these women attended the first ultrasound screening at 12 + 0–13 + 6 weeks + days of gestation in one of the ten hospital maternity clinics participating in the study. The cohort profile^30^ contains details of the study design and inclusion criteria.

### Ethics

The study protocol was approved by the Ethical Committees of the Helsinki and Uusimaa Hospital District and by the participating hospitals. A written informed consent was obtained from all women.

### Maternal characteristics

We tested 10 different maternal environments:

### Depressive symptoms

Starting from 12 + 0–13 + 6 gestational weeks + days pregnant women filled in the 20 item Center for Epidemiological Studies Depression Scale (CES-D)^59^ for depressive symptoms in the past 7 days. They filled in the CES-D scale biweekly until 38 + 0–39 + 6 weeks + days of gestation or delivery. We used the mean-value across all the CES-D measurements.

### Symptoms of anxiety

At 12 + 0–13 + 6 weeks + days of gestation, women filled in the 20 item Spielberger’s State Trait Anxiety Inventory (STAI)^60^ for anxiety symptoms in the past 7 days. They filled in the STAI scale biweekly until 38 + 0–39 + 6 weeks + days of gestation or delivery. We used the mean-value across all these measurements.

### Betamethasone

Antenatal betamethasone treatment (yes/no) was derived from the hospital records and the Finnish Medical Birth Register (MBR).

### Delivery method

Mode of delivery (vaginal delivery vs. caesarean section) was derived from patient records and MBR.

### Parity

Parity (number of previous pregnancies leading to childbirth) at the start of present pregnancy was derived from the hospital records and the MBR.

### Maternal age

Maternal age at delivery (years) was derived from the hospital records and the MBR.

### Pre-pregnancy BMI

Maternal pre-pregnancy BMI (kg/m^2^), calculated from measurements weight and height verified at the first antenatal clinic visit at 8 + 4 (SD 1 + 3) gestational week was derived from the hospital records and the MBR.

### Hypertension

Hypertension was defined as any hypertensive disorder including gestational hypertension, chronic hypertension and preeclampsia against normotension. Gestational hypertension was defined as systolic/diastolic blood pressure ≥140/90 mm Hg on ≥2 occasions at least 4 h apart in a woman who was normotensive before 20th week of gestation. Preeclampsia was defined as systolic/diastolic blood pressure ≥140/90 mm Hg on ≥2 occasions at least 4 h apart after 20th week of gestation and proteinuria ≥300 mg/24 h. Chronic hypertension was defined as systolic/diastolic blood pressure ≥140/90 mm Hg on ≥2 occasions at least 4 h apart before 20th gestational week or medication for hypertension before 20 weeks of gestation.

### Gestational diabetes and oral glucose tolerance test

Gestational diabetes was defined as fasting, 1 h or 2 h plasma glucose during a 75 g oral glucose tolerance test ≥5.1, ≥10.0 and/or ≥8.5 mmol/L, respectively, that emerged or was first identified during pregnancy. We took the area under the curve from the three measurements as a single measure for the oral glucose tolerance test (OGTT) itself.

### Genotyping and imputation

Genotyping was performed on Illumina Human Omni Express Exome Arrays containing 964,193 SNPs. Only markers with a call rate of at least 98%, a minor allele frequency of at least 1% and a *p*-value for deviation from Hardy-Weinberg-Equilibrium >1.0 × 10^−06^ were kept in the analysis. After QC, 587,290 SNPs were available.

In total, 996 cord blood samples were genotyped. Samples with a call rate below 98% (*n* = 11) were removed.

Any pair of samples with IBD estimates >0.125 was checked for relatedness. As we corrected for admixture in our analyses using MDS-components (see Supplementary Fig. 10), these samples were kept except for one pair which could not be resolved. From this pair we excluded one sample from further analysis. Individuals showing discrepancies between phenotypic and genotypic sex (*n* = 1) were removed. We also checked for heterozygosity outliers but found none. Nine hundred and eighty-three participants were available in the final dataset.

Before imputation, AT and CG SNPs were removed. Imputation was performed using shapeit2 (http://mathgen.stats.ox.ac.uk./genetics_software/shapeit/shapeit.html) and impute2 (https://mathgen.stats.ox.ac.uk/impute/impute_v2.html). Chromosomal and base pair positions were updated to the 1000 Genomes Phase 3 reference set, allele strands were flipped where necessary.

After imputation, we reran quality control, filtering out SNPs with an info score <0.8, a minor allele frequency below 1% and a deviation from HWE with a *p*-value <1.0 × 10 ^-06^.

This resulted in a dataset of 9,402,991 SNPs. After conversion into best guessed genotypes using a probability threshold of 90%, we performed another round of QC (using SNP-call rate of least 98%, a MAF of at least 1% and a *p*-value threshold for HWE of 1.0 × 10^−06^), after which 7,314,737 SNPs remained for the analysis.

For the evaluation of which model best explained the methylation sites, we pruned the dataset using a threshold of *r*^2^ of 0.2 and a window-size of 50 SNPs with an overlap of 5 SNPs. The final, pruned dataset contained 788,156 SNPs. 36,241 of these variants were DeepSea variants (see Methods below).

### DNA methylation

Cord blood samples were run on Illumina 450k Methylation arrays. The quality control pipeline was set up using the R-package *minfi*^61^ (https://www.r-project.org). Three samples were excluded as they were outliers in the median intensities. Furthermore, 20 samples showed discordance between phenotypic sex and estimated sex and were excluded. Nine samples were contaminated with maternal DNA according to the method suggested by Morin et al.^62^ and were also removed.

Methylation beta-values were normalised using the *funnorm* function^63^. After normalisation, two batches, i.e., slide and well, were significantly associated and were removed iteratively using the *Combat* function^64^ in the *sva* package^65^.

We excluded any probes on chromosome X or Y, probes containing SNPs and cross-hybridising probes according to Chen et al.^53^ and Price et al.^66^ Furthermore, any CpGs with a detection *p*-value >0.01 in at least 25% of the samples were excluded.

The final dataset contained 428,619 CpGs and 822 participants. For 817 of these, also genotypes were available.

An additional 161 cord blood samples were run on Illumina EPIC Methylation arrays.

Three samples were excluded as they were outliers in the median intensities. Three samples showed discordance between phenotypic sex and estimated sex and were excluded. Three samples were contaminated with maternal DNA and were also removed^62^.

Methylation beta-values were normalised using the *funnorm* function^63^ in the R*–*package *minfi*^61^. Three samples showed density artefacts after normalisation and were removed from further analysis. We excluded any probes on chromosome X or Y, probes containing SNPs and cross-hybridising probes according to Chen et al.^53^, Price et al.^66^ and McCartney et al.^67^. Furthermore, any CpGs with a detection *p*-value >0.01 in at least 25% of the samples were excluded. The final dataset contains 812,987 CpGs and 149 samples. After normalisation no significant batches were identified. For 146 of these samples, genotypic data was also available.

Cord blood cell counts were estimated for seven cell types (nucleated red blood cells, granulocytes, monocytes, natural killer cells, B cells, CD4(+)T cells, and CD8(+)T cells) using the method of Bakulski et al.^68^ which is incorporated in the R-package *minfi*^61^.

### Identification of VMRs (variable methylated regions)

The VMR approach was described by Ong and Holbrook^54^. We chose all 42,862 CpGs with a MAD score greater than the 90^th^ percentile. For each CpG-site, the MAD (median absolute deviation) is defined as the median of the absolute deviations from each individual’s methylation beta-value at this CpG-site to the CpG’s median. A candidate VMR region was defined as at least two spatially contiguous probes which were at most 1 kb apart of each other. This resulted in 3982 VMRs in the 450 K samples and in 8547 VMRs in the EPIC sample. The CpG with the highest MAD scores was chosen as representative of the whole VMR in the statistical analysis.

### The Drakenstein cohort

Details on this cohort and the assessed phenotypes can be found in refs. ^34,35^. The birth cohort design recruits pregnant women attending one of two primary health care clinics in the Drakenstein sub-district of the Cape Winelands, Western Cape, South Africa – Mbekweni (serving a black African population) and TC Newman (serving a mixed ancestry population). Consenting mothers were enroled during pregnancy, and mother–child dyads are followed longitudinally until children reach at least 5 years of age. Mothers are asked to request that the father of the index pregnancy attend a single antenatal study visit where possible. Follow-up visits for mother–child dyads take place at the two primary health care clinics and at Paarl Hospital.

Pregnant women were eligible to participate if they were 18 years or older, were accessing one of the two primary health care clinics for antenatal care, had no intention to move out of the district within the following year, and provided signed written informed consent. Participants were enroled between 20 and 28 weeks’ gestation, upon presenting for antenatal care visit. In addition, consenting fathers of the index pregnancy when available were enroled in the study and attended a single antenatal study visit.

### Ethics

The study was approved by the Faculty of Health Sciences, Human Research Ethics Committee, University of Cape Town (401/2009), by Stellenbosch University (N12/02/0002), and by the Western Cape Provincial Health Research committee (2011RP45). All participants provided written informed consent.

### Maternal characteristics

After providing consent, participants were asked to complete a battery of self-report and clinician-administered measures at a number of antenatal and postnatal study visits. All assessed phenotypes are described in detail in ref. ^34^. Here, we give a short outline on the phenotypes which were used in our analysis. Maternal parity was obtained from the antenatal record; maternal age was from the date of birth as recorded on the mothers’ national identity document. The mode of delivery was ascertained by direct observation of the birth by a member of the study team as all births occurred at Paarl hospital. The SRQ-20^69^ is a WHO-endorsed measure of psychological distress consisting of 20 items which assess non-psychotic symptoms, including symptoms of depressive and anxiety disorders. Each item is scored according to whether the participant responds in the affirmative (scored as 1) or negative (scored as 0) to the presence of a symptom. Individual items are summed to generate a total score. The Beck Depression Inventory (BDI-II) is a widely-used and reliable measure of depressive symptoms^70^. The BDI-II comprises 21 items, each of which assesses the severity of a symptom of major depression. Each item is assessed on a severity scale ranging from 0 (absence of symptoms) to 3 (severe, often with functional impairment). A total score is then obtained by summing individual item responses, with a higher score indicative of more severe depressive symptoms.

Smoking was assessed using The Alcohol, Smoking and Substance Involvement Screening Test (ASSIST)^71^, a tool that was developed by the WHO to detect and manage substance use among people attending primary health care services. The tool assesses substance use and substance-related risk across 10 categories (tobacco, alcohol, cannabis, cocaine, amphetamine-type stimulants, inhalants, sedatives/sleeping pills, hallucinogens, opioids and other substances), as well as enquiring about a history of intravenous drug use. Total scores are obtained for each substance by summing individual item responses, with a higher score indicative of greater risk for substance-related health problems.

Hypertension was assessed by blood pressure measured antenatally.

### Genotyping and Imputation

Genotyping in DCHS was performed using the Illumina PsychArray for those samples with 450k data, or the Illumina GSA for those samples with EPIC DNA methylation data (Illumina, San Diego, USA). For both array types, QC and imputation was the same; first, raw data was imported into Genome Studio and exported into R for QC. SNPs were filtered out if they had a tenth percentile GC score below 0.2 or an average GC score below 0.1, for a total of 140 SNPs removed. Phasing was performed using shapeit, and imputation was performed using impute2 with 1000 Genomes Phase 1 reference data. After imputation, we used qctool to filter out SNPs with an info score <0.8 or out of Hardy–Weinberg equilibrium. All SNPs with MAF <1% were removed.

As after imputation, only 5286 DeepSEA variants were available for those samples genotyped on the PsychArray and only 4049 for those samples genotyped on the GSAchip, we performed LD-pruning based on a threshold of *r*^2^ of 0.2 and a window-size of 50 SNPs with an overlap of 5 SNPs. This resulted in 162,292 SNPs (PsychArray) and 176,553 SNPs (GSAchip).

### DNA methylation

We performed basic quality control on data generated by either the 450k or EPIC arrays using Illumina’s Genome Studio software for background subtraction and colour correction. Data was filtered to remove CpGs with high detection p values, those on the X or Y chromosome, or with previously identified poor performance. 450k data was normalised using SWAN and EPIC data using BMIQ, and both used ComBat to correct for chip (both), and row (450k only). Details for DNA methylation measurements and quality control have been published^62^. The final analysis was performed with 107 samples with methylation levels from the 450k array and 151 with methylation levels assessed on the EPIC array and available genotypes. Neonatal blood cell counts were estimated for seven cell types: nucleated red blood cells, granulocytes, monocytes, natural killer cells, B cells, CD4(+)T cells, and CD8(+)T cells^68^.

### VMRs

We identified 6072 candidate VMRs in DCHS I and 10,005 candidate VMRs in DCHS II.

### The UCI cohort

Mothers and children were part of an ongoing, longitudinal study, conducted at the University of California, Irvine (UCI), for which mothers were recruited during the first trimester of pregnancy^31–33^. All women had singleton, intrauterine pregnancies. Women were not eligible for study participation if they met the following criteria: corticosteroids, or illicit drugs during pregnancy (verified by urinary cotinine and drug toxicology). Exclusion criteria for the newborn were preterm birth (i.e., less than 34 weeks of gestational age at birth), as well as any congenital, genetic, or neurologic disorders at birth.

### Ethics

The UCI institutional review board approved all study procedures and all participants provided written informed consent.

### Maternal characteristics

Maternal sociodemographic characteristics (age, parity) were obtained via a standardised structured interview at the first pregnancy visit. Maternal pre-pregnancy BMI (weight kg/height m^2^) was computed based on pre-pregnancy weight abstracted from the medical record, and maternal height was measured at the research laboratory during the first pregnancy visit. Obstetric risk conditions during pregnancy, including presence of gestational diabetes and hypertension, and delivery mode were abstracted from the medical record. At each pregnancy visit the Center for Epidemiological Studies Depression Scale^59^ and the State scale from the State–Trait Anxiety Inventory^60^ were administered. For individuals with <3 missing items on any scale at any time point, the mean responses for that scale were calculated and then multiplied by the total number of items in the respective scale, to generate total scale scores that are comparable to those generated from participants without any missing data. We used the average depression and anxiety score throughout pregnancy in the calculations. Maternal smoking during pregnancy was determined by maternal self-report and verified by measurement of urinary cotinine concentration. Urinary cotinine was assayed in maternal samples collected at each trimester using the Nicotine/COT(Cotinine)/Tobacco Drug Test Urine Cassette (http://www.meditests.com/nicuintescas.html), which involves transferring 4 drops of room temperature urine into the well of the cassette, and employs a cutoff for COT presence of 200 ng/ml. Endorsement of smoking or detection of urinary COT in any trimester was coded as 1, and absence of evidence for smoking in any trimester coded as 0.

### Genotyping

Genomic DNA was extracted from heel prick blood samples and used for all genomic analysis. Genotyping was performed on Illumina Human Omni Express (24 v1.1) Arrays containing 713,014 SNPs. All samples had a high call rate (above 97%). SNPs with a minor allele frequency >5% and a p-value for deviation from Hardy-Weinberg-Equilibrium >1.0 × 10^−25^ were retained for analysis. After QC, 602,807 SNPs were available.

### Imputation

Before imputation, chromosomal and base pair positions were updated to the Haplotype Reference Consortium (r1.1) reference set, allele strands were flipped where necessary. Phasing was performed using EAGLE2 (https://data.broadinstitute.org/alkesgroup/Eagle/) and imputation was performed using PBWT (https://github.com/VertebrateResequencing/pbwt). Imputed SNPs with an info score <0.8, duplicates and ambiguous SNPs were removed resulting in 21,341,980 SNPs. All SNPs with MAF <0.01 were removed. Of the remaining SNPs, 19,530 were DeepSEA variants.

### DNA methylation

DNAm analysis using the Infinium Illumina MethylationEPIC BeadChip (Illumina, Inc., San Diego, CA) was performed according to the manufacturer´s guidelines in using genomic DNA derived from neonatal heel prick samples. Quality Control carried out in *minfi*^61^. No outliers were detected in the median intensities of methylated and unmethylated channels. All samples had a high call rate of at least 95% and their predicted sex was the same as the phenotypic sex. We removed CpGs with a high detection value (*p* < 0.0001), probes missing >3 beads in >5% of the cohort, in addition to non-specific/cross-hybridising and SNP probes^66,67^. Methylation beta-values were normalised using functional normalisation (*funnorm*)^63^. We also iteratively adjusted the data for relevant technical factors, i.e., array row, experimental batch and sample plate, using *Combat*^64^. The final dataset contained 768,910 CpGs. Neonatal blood cell counts were estimated for seven cell types: nucleated red blood cells, granulocytes, monocytes, natural killer cells, B cells, CD4(+)T cells, and CD8(+)T cells^68^. The final dataset contained 121 samples with available genotypes and methylation values.

### VMRs

Applying the same procedure as for PREDO I and PREDO II, we identified 9525 candidate VMRs in the ICU cohort.

### The MoBa cohort

Participants represent two subsets of mother-offspring pairs from the national Norwegian Mother and Child Cohort Study (MoBa)^72^. MoBa is a prospective population-based pregnancy cohort study conducted by the Norwegian Institute of Public Health. The years of birth for MoBa participants ranged from 1999 to 2009. MoBa mothers provided written informed consent. Each subset is referred to here as MoBa1 and MoBa2. MoBa1 is a subset of a larger study within MoBa that included a cohort random sample and cases of asthma at age 3 years^73^. We previously reported an association between maternal smoking during pregnancy and differential DNA methylation in MoBa1 newborns^74^. We subsequently measured DNA methylation in additional newborns (MoBa2) in the same laboratory (Illumina, San Diego, CA)^11^. MoBa2 included cohort random sample plus cases of asthma at age 7 years and non-asthmatic controls. Years of birth were 2002–2004 for children in MoBa1, 2000–2005 for MoBa2.

### Ethics

The establishment and data collection in MoBa obtained a license from the Norwegian Data Inspectorate and approval from The Regional Committee for Medical Research Ethics. Both studies were approved by the Regional Committee for Ethics in Medical Research, Norway. In addition, MoBa1 and MoBa2 were approved by the Institutional Review Board of the National Institute of Environmental Health Sciences, USA.

### Maternal characteristics

To replicate specific GxE and G + E from PREDO I, we focused on those characteristics which were available in both cohorts: maternal age, pre-pregnancy BMI and hypertension.

Within MoBa, the questionnaires at weeks 17 and 30 include general background information as well as details on previous and present health problems and exposures. The birth record from the Medical Birth Registry of Norway^75^ which includes maternal health during pregnancy as well as procedures around birth and pregnancy outcomes, is integrated in the MoBa database.

### Genotyping and imputation

DNA was extracted from the MoBa biobank and genotyped on the Illumina HumanExomeCore platform. The genotypes were called with GenomeStudio software. Phasing and imputation were done using shapeit2 (https://mathgen.stats.ox.ac.uk./genetics_software/shapeit/shapeit.html) and impute2 (https://mathgen.stats.ox.ac.uk/impute/impute_v2.html) with the thousand genomes phase 3 reference panel for the European population. Variants with a imputation score of <0.8 and with a minor allele frequency below 1% were filtered out.

### DNA methylation

Details of the DNA methylation measurements and quality control for the MoBa1 participants were previously described^36^ and the same protocol was implemented for the MoBa2 participants. Briefly, at birth, umbilical cord blood samples were collected and frozen at birth at −80 °C. All biological material was obtained from the Biobank of the MoBa study^36^. Bisulfite conversion was performed using the EZ-96 DNA Methylation kit (Zymo Research Corporation, Irvine, CA) and DNA methylation was measured at 485,577 CpGs in cord blood using Illumina’s Infinium HumanMethylation450 BeadChip^76^. Raw intensity (.idat) files were handled in R using the *minfi* package to calculate the methylation level at each CpG as the beta-value (*β* = intensity of the methylated allele (M)/(intensity of the unmethylated allele (U)+ intensity of the methylated allele (M) + 100)) and the data was exported for quality control and processing. Control probes (*N* = 65) and probes on X (*N* = 11 230) and Y (*N* = 416) chromosomes were excluded in both datasets. Remaining CpGs missing >10% of methylation data were also removed (*N* = 20 in MoBa1, none in MoBa2). Samples indicated by Illumina to have failed or have an average detection *p* value across all probes <0.05 (*N* = 49 MoBa1, *N* = 35 MoBa2) and samples with gender mismatch (*N* = 13 MoBa1, *N* = 8 MoBa2) were also removed. For MoBa1 and MoBa2, we accounted for the two different probe designs by applying the intra-array normalisation strategy Beta Mixture Quantile dilation (BMIQ)^77^. The Empirical Bayes method via *ComBat* was applied separately in MoBa1 and MoBa2 for batch correction using the *sva* package in R^65^. After quality control exclusions, the sample sizes were 1068 for MoBa1 and 685 for MoBa2.

After QC, the total number of samples was 1732, with 1592 overlapping with the methylation samples. Specific G + E and GxE associations were calculated in the combined dataset of MoBa1 and MoBa2, while VMR analysis was conducted in MoBa1 only.

### Regression analysis

Linear regression analysis was conducted using the *lm* function in R 3.3.1 (https://www.r-project.org). We included the child’s sex, gestational age, seven estimated cell counts as well as the first two (PREDO I and PREDO II), first three (UCI) and first five (DCHS I and II) principal components of the MDS analysis on the genotypes in the model. The corresponding plot of the first ten MDS-components in PREDO is depicted in Figure S4. SNP genotypes were recoded into a count of 0, 1 or 2 representing the number of minor allele copies. For each VMR site, we tested SNPs located in a 1MB window up- and downstream of the specific site. In PREDO and UCI, we restricted the analysis to DeepSEA variants while we used the pruned SNP-set in DCHS.

For each VMR, we tested four models:

1. Methylation at tagCpG ~ covariates + environment
2. Methylation at tagCpG ~ covariates + SNP
3. Methylation at tagCpG ~ covariates + SNP + environment
4. Methylation at tagCpG ~ covariates + SNP + environment + SNP×environment

In model (1) we included all ten different environments, in model (2) all DeepSEA cis SNPs and in models (3) and (4) all possible environment-cis-SNP combinations. Please also see Fig. 1.

For each model, the AIC, Akaike’s information criterion^37^ was calculated and the model with the lowest AIC was chosen as the best model. The AIC was obtained using the *AIC* function in R 3.3.1 (https://www.r-project.org).

*P*-values were obtained from the summary function and adjusted for the number of tested Es (E model), of tested cis SNPs (G model) or of tested cis SNP-environment combinations (G + E/GxE model) using Bonferroni-correction. Afterwards, we used FDR to correct for all tested tagCpGs (all models) using *p.adjust* in R.

### Enrichment analyses

With regard to enrichment for VMRs, CpG-site within VMRs were compared to all other CpG-sites on the 450 K array located in non-VMR-regions. With regard to enrichment for VMRs best explained by G, G + E or GxE, tagCpGs best explained by the specific model were compared to tagCpGs best explained by any of the other models. For enrichment tests for DeepSEA SNPs, non-DeepSEA SNPs present in our dataset were used as comparison group. Enrichment tests were performed based on a hyper-geometric test, i.e. a Fisher-test. The significance levels was set at *p* < 0.05.

With regard to enrichment for GWAS hits, DeepSEA variants were matched to GWAs variants based on chromosome and position (hg19). To check for enrichment for nominal significant GWAS hits, the full summary statistics were derived from the respective publication.

Histone ChiP-seq peaks from Roadmap Epigenomics project for blood and embryonic stem cells were downloaded from http://egg2.wustl.edu/roadmap/data/byFileType/peaks/consolidated/broadPeak/.

The pre-processed consolidated broad peaks from the uniform processing pipeline of the Roadmap project were used.

### Genomic annotation mapping

CpG sites were mapped to the genome location according to Illumina’s annotation using the R-package *minfi*.

### DeepSEA analysis

Pretrained DeepSEA model was downloaded from:

http://deepsea.princeton.edu/media/code/deepsea.v0.94.tar.gz and variant files in VCF format are used for producing e-values. VCF files were first split into smaller files each containing one million variants and the model was run using the command line on a server with a NVIDIA Titan X GPU card.

We reran our models using only DeepSEA variants which had been identified by the algorithm of Zhou and Troyanskaya^38^. This method predicts functionality of a SNP based on the DNA-sequence. We included all 212,210 variants with a functional significance e-value below 5 × 10^−05^. The e-values represent the significance of the regulatory impact of given variants compared to one million random variants.

### Random-effects meta-analysis

GxE and G + E result for PREDO and for MoBa were meta-analysed using a random-effects model in the R-package rmeta. Replication was defined as DeepSEA-tagCpG-environment combinations showing the same effect direction in both cohorts, presenting with smaller *p*-values as for PREDO alone and with a FDR-corrected *p*-value (across all combinations tested in the meta-analysis) below 0.05.

### Reporting summary

Further information on research design is available in the Nature Research Reporting Summary linked to this article.

## Supplementary information

Supplementary Information

Peer Review File

Description of Additional Supplementary Files

Supplementary Data 1

Supplemental Data 2

Supplemental Data 3

Supplemental Data 4

Supplemental Data 5

Supplemental Data 6

Supplemental Data 7

Supplemental Data 8

Supplemental Data 9

Supplemental Data 10

Supplemental Data 11

Supplemental Data 12

Supplemental Data 13

Supplemental Data 14

Supplemental Data 15

Supplemental Data 16

Supplemental Data 17

Supplemental Data 18

Reporting Summary

## Data Availability

Due to ethical issues and consent the datasets analysed during the current study are not publicly available. However, an interested researcher can obtain a de-identified dataset after approval from the PREDO Study Board. Data requests may be subject to further review by the national register authority and by the ethical committees. Data can be obtained upon reasonable request from the PREDO Study Board (predo.study@helsinki.fi) or individual researchers. The summary statistics of the best models for PREDO I are accessible at: https:/doi.org/10.6084/m9.figshare.8074964. For access to the UCI cohort, please contact claudia.buss@charite.de, for access to DCHS please contact Heather.Zar@uct.ac.za, for MoBa access please apply for data access at https:/www.fih.no
