# Supplementary material for: Integrated analysis of environmental and genetic influences on cord blood DNA methylation in new-borns
Source: Nat Commun. 2019 Jun 11;10:2548. doi: 10.1038/s41467-019-10461-0 (PMC6559955; doi:10.1038/s41467-019-10461-0)
Supplement: Supplementary file 1 — Supplementary Information [file 41467_2019_10461_MOESM1_ESM.pdf]

Integrated analysis of environmental and genetic influences on cord blood DNA methylation in new-borns

Czamara et al.

Supplementary Notes

Supplementary Note 1

Variably methylated regions

While overall methylation levels at CpGs are bimodally distributed with peaks at very low methylation (beta-value < 0.1) and very high methylation (beta-value > 0.8), the distribution of methylation at CpGs in VMRs is unimodal and VMRs presented with intermediate methylation levels (median beta-value=0.52, 25th-75th percentile = 0.39-0.62, see Supplementary Figure 1A). As compared to all qc-ed CpG-sites on the 450K array, VMRs in cord blood were enriched for OpenSea ($p=5.26 \times 10^{-03}$, OR=1.06, Fisher-test) and Shores ($p=1.64 \times 10^{-90}$, OR=1.55, Fisher-test) and depleted for Islands ($p=4.35 \times 10^{-32}$, OR=0.77, Fisher-test). Furthermore, VMRs were enriched for distal intergenic regions ($p=4.64 \times 10^{-31}$, OR=1.29, Fisher-test) and depleted for introns ($p=2.53 \times 10^{-07}$, OR=0.90, Fisher-test). Additionally, we checked if VMRs were enriched for transcription factor (TF) binding sites. Using the data of ReMap [1] and ENCODE [2] we found significant enrichment of these sites for VMRs ($p=1.21 \times 10^{-60}$, OR=1.50, Fisher-test) as compared to non-VMRs on the 450K array. Eighty-two % of all VMR-CpGs overlapped with at least one TF, with CTCF and NR3C3 being among the top enriched factors.

VMRs have been associated with specific chromatin states [3]. As compared to non-VMRs, VMRs in our dataset were depleted for active and flanking transcription start sites (TSS), for strong transcription and for transcription at 5' and 3'. In contrast, VMRs were enriched for weak transcription, enhancers, ZNF genes and repeats, heterochromatin, bivalent/poised TSS, bivalent flanking TSS/enhancers, bivalent enhancers, repressed and weak repressed PolyComb sites (Supplementary Figure 1B).

We used the publicly available eQTM results from Bonder et al. [4], who examined whether VMRs significantly overlapped with expression quantitative trait methylation sites (eQTMs),

i.e., CpGs significantly associated with gene expression. As the analysis presented by Bonder et al. was based on CpGs located in proximity to the TSS of the specific transcript, we used only PREDO I CpGs located in VMRs and also located within genes (n=5,905). The overlap between significantly associated eQTMs from Bonder et al. and VMRs in PREDO I was significantly higher than expected by chance ($p=9.99 \times 10^{-05}$, based on sampling of 10,000 random CpG-sets), revealing that areas with high levels of inter-individual variation in DNA methylation overlap strongly with sites associated with gene expression.

Additionally, 6,074 CpG-sites previously associated with maternal smoking [5] and 104 CpG-sites previously associated with maternal BMI [6] significantly overlapped (p=0.009 for smoking and p=0.0009 for BMI, based on sampling of 1,000 random CpGs-sets) with VMR CpGs. Furthermore, CpG-sites reported in [5,6] showed higher MAD-scores in PREDO I as compared to CpGs which were not significantly associated in either of these studies.

Supplementary Note 2

Evaluation of next best models

We also evaluated the respective next best models, i.e. the models presenting with the second smallest AIC. If the best model was G (n=1,194), the next best model was G+E in 70% of the cases (n=840), and GxE for the remaining part (n=354). For the 1,616 tagCpGs where the best model was GxE, the next best model was mostly G+E (n=868) followed by the G model (n=748). In the case of the 1,171 tag CpGs with best model G+E, the next best model was mostly GxE (n=750), followed by G only (n=421). Interestingly, E never occurred as next best model. For the one CpG with best model E, the next best model was G+E. The delta AIC for best model GxE to the next best model was significantly higher (mean delta AIC=2.38) as compared to CpGs with G as the best model (mean delta AIC=0.89, $p= 2.22 \times 10^{-96}$, Wilcoxon-test) or G+E as the best model (mean delta AIC=0.98, $p=4.78 \times 10^{-80}$,

Wilcoxon-test). The delta AIC for best model G+E (mean=0.98) was also significantly higher as compared to best model G (mean=0.89, p=2.58 x 10^{-03}, Wilcoxon-test).

Supplementary Note 3

DeepSEA prediction of SNP function

Again here, we observed that the delta AIC for best model GxE to the next best model was significantly higher (mean delta AIC=2.18) as compared to CpGs with G as the best model (mean delta AIC=0.89, p= 4.57 x 10^{-70}, Wilcoxon-test) or G+E as the best model (mean delta AIC=0.94, p=8.81 x 10^{-63}). Furthermore, 24.8% of tagCpGs best explained by the G+E model were associated with maternal betamethasone treatment), 41.2% with general maternal factors (mostly maternal age) and 34.00 % with factors related to metabolism (pre-pregnancy BMI, hypertension, gestational diabetes). For best model GxE, the proportions were similar with 21.6%, 45.4% and 33.0%, respectively.

Supplementary Note 4

Is the proportion of best models dependent on the variability of CpG-sites?

The power to detect meQTLs depends on the variance of DNA methylation at the specific CpG-site [7] and the allele frequency of SNPs mapped in close proximity to the most variably methylated sites [8]. Therefore, VMRs, which by definition are restricted to CpG-sites with a high variability, might not only be biased for the identification of significant meQTLs but also have a higher proportion of the variance explained by G using the AIC. We investigated if this was true by re-running the E, G, G+E and GxE models on all CpGs-sites, regardless of whether they were located in VMRs or not. As maternal age was one of the most important predictors for methylation levels at VMRs in our analysis (see Figure 2B), we focused on this phenotype. We found that, across all CpG-sites and all variability levels (see Supplementary Figure 2), the pattern of best models remained stable indicating that, at least in our sample,

combined G and E effects are also present in sites not located in VMRs and in less variable sites.

Supplementary Note 5

Is the proportion of best models dependent on environment?

Up to now, we chose the best model with regard to a multitude of different prenatal phenotypes. We next investigated if the same pattern of best models is observed across the different environmental phenotype by determining the best model (E , G, G+E and GxE) for each phenotype independently. We did not observe high correlations between the different investigated prenatal phenotypes (see Supplementary Figure 4A). The strongest correlation was present between anxiety and depression scores (Pearson's correlation coefficient r=0.86) followed by the area under the curve (AUC) of the oral glucose tolerance test (ogtt) and gestational diabetes (r=0.69). All other correlations were below 0.40. In this analysis, we observed substantial differences of the relative impact of G and E on DNA methylation when stratifying by different types of prenatal phenotypes. In fact, maternal age and betamethasone treatment show the highest proportion of VMRs with the best models G+E and GxE (about 25%), while other prenatal factors had significantly less of the best G+E or GxE models (see Supplementary Figure 4B). This analysis suggests that as expected, different types of exposures or maternal factors have different relative impact on DNA methylation.

Supplementary Note 6

Functional annotation of VMRs with different best models

TagCpGs best explained by GxE showed a trend for enrichment for OpenSea (p=0.10, OR=1.18, Fisher-test), while tagCpGs best explained by E were depleted for Islands (p= 2.51 x 10^{-02}, OR=0.46, Fisher-test, Figure 3B). Furthermore, G+E tagCpGs were depleted for promoters (p=1.12 x 10^{-02}, OR=0.82, Fisher-test , Figure 3C).

With regard to enrichment/depletion of specific histone marks based on the ENCODE data [2] in comparison to all tagCpGs, E tagCpGs were depleted for flanking active TSS ($p=1.09x10^{-02}$, OR=0.46, Fisher-test). G tag CpGs were depleted for bivalent TSS/enhancers ($p=3.77x10^{-02}$, OR=0.83, Fisher-test) and enriched for weak TSS ($p=4.80x10^{-02}$, OR=1.15, Fisher-test) and for enhancers ($p=4.12x10^{-02}$, OR=1.15, Fisher-test). G+E tagCpGs were enriched for heterochromatin ($p=3.21x10^{-02}$, OR=1.29, Fisher-test), for flanking bivalent TSS/enhancers ($p=1.77x10^{-02}$, OR=1.25, Fisher-test), for bivalent enhancers ($p=7.46x10^{-02}$, OR=1.25, Fisher-test) and for repressed PolyComb ($p=2.98x10^{-02}$, OR=1.17, Fisher-test, see figure 4A and B).

Supplementary Note 7

Replication of best models in independent cohorts

As after imputation only few DeepSEA variants were available for the DCHS cohort, we performed LD-pruning in this cohort and ran the analysis on the pruned SNP set (see Methods). In all these cohorts, we observed the same distribution of median methylation levels at VMR CpG-sites as in PREDO I: while overall methylation levels at CpGs were bimodally distributed as expected, the distribution of methylation levels at CpGs within VMRs was unimodal and VMRs presented with intermediate methylation levels (see Supplementary Figure 6). The length of VMRs was similar across cohorts with an overall mean of 3.8 CpGs and individual means: PREDO I=3.26, PREDO II=3.36, DCHS I=3.93, DCHS II=3.66, UCI=3.57.

Supplementary Note 8

Association with smoking

As we did not observe significant main E effects on DNA methylation for most of the tested Es in our cohorts, we chose to rerun the analyses focusing on maternal smoking, described as one of the most highly replicated factors shaping the newborns' methylome [5]. This would

allow an assessment of how inclusion of a validated E factor would influence the relative distribution of the best models. We first ran traditional epigenome-wide association analyses for maternal smoking in the cohorts where this exposure was included, namely the UCI, DCHS I and DCHS II. We observed that those CpG-sites where association with smoking was nominally significant in our samples, were significantly enriched for CpG-sites which had been reported to be associated with this exposure in the meta-analysis by Joubert et al. [5] at an FDR corrected p-value cut-off of 0.05 (UCI, $p=1.27\times10^{-27}$, OR=1.77, DCHS I, $p=7.20\times10^{-33}$, OR=1.81, DCHS II $p=1.49\times10^{-17}$, OR=1.60, Fisher-tests). Next, we tested whether those CpGs that were associated with maternal smoking in Joubert et al. [5] at FDR 0.05, presented with best model E=smoking, or if the inclusion of genotype yielded a better model. For UCI 5,362 CpGs out of the 6,073 reported CpGs were available. From these, 26 (<1 %) were best explained by smoking alone (E), whereas 5,044 (94.1%) were best explained by genotype (G), 126 (2.3%) by G+maternal smoking (G+E) and 166 (3.1%) by Gxmaternal smoking (GxE). In DCHS I, 5633 of the top CpGs were available, 4,723 (83.9%) presented with best model G, 639 (11.3%) with best model GxE and 271 (4.8 %) with best model G+E. In DCHS II, out of 5,405 CpGs, 2 (< 1%) presented with best model E, 3,072 (56.8%) with best model G, 1,635 (30.2%) with best model GxE and 696 (12.9%) with best model G+E. This underscores the point that even for phenotypes, such as maternal smoking, with documented main E effects on cord blood methylation, genotypic information should be considered. This is further strengthened by our analysis within the MoBa cohort (n=1,023) which contains a higher amount of smoking pregnant women (n=148) than the other cohorts. In this cohort, we observed that 10% of tagCpGs were best explained by maternal smoking. For 40% of these, the next best model based on the AIC is again model G.

Supplementary Note 9

Validation of specific GxE and G+E combinations

Although underpowered to robustly detect significant GxE interactions, we examined if specific combined effects of genotype and environment (and not just sharing a similar best model via AIC) in PREDO I, could be replicated in an additional independent sample, the MoBa cohort. We restricted the analysis to those combinations of CpG-DeepSEA SNP and environments that were nominally significant for GxE or G+E and also presented with the lowest AIC for this specific model in PREDO I. Of these, 515 GxE and 178 G+E combinations were also available in the MoBa cohort. We combined both studies via random-effects-meta-analyses where 6 GxE and 18 G+E combinations were significantly associated and survived multiple testing across all tested combinations (FDR 0.05), showing the same direction of effects in both cohorts and presenting with lower p-values in the meta-analysis as compared to PREDO I alone. These results are depicted in Supplementary Data 17 and 18. Two of the tophits for GxE and G+E are presented in Supplementary Figure 9A and B, respectively.

Supplementary Tables

Supplementary Table 1: tagCpGs with best model E in pruned PREDO I dataset

CpG	Chr	bp_CpG	E	AIC_E	p_E
cg18561676	1	148855262	parity	-1865,57	5,88E-03

bp_CpG: genomic position of CpG in base-pairs (hg19)
E: environment included in E model
AIC_E: AIC of E model
p_E: nominal p-value of E effect

Supplementary Discussion

Some differences between our study and the study of Teh at al. [9] should be mentioned. First, our sample was larger and also ethnically more homogenous as compared to Teh at al. who studied a mixed Asian population. Second, not all of the environmental factors that were tested in Teh et al. were available for our cohort. Third, we used imputed SNP genotypes whereas Teh et al. used only measured genotypes. In fact, 64% of the SNPs that are involved in best G models in our dataset were imputed. Fourth, as compared to Teh et al., we looked into a different set of CpGs as VMRs between the PREDO I and the Teh et al studies only showed a slight overlap (n = 219 tagCpGs), likely due to differences in tissue and ethnicity.

Supplementary References

1 Griffon, A. *et al.* Integrative analysis of public ChIP-seq experiments reveals a complex multi-cell regulatory landscape. *Nucleic Acids Res* **43**, e27, doi:10.1093/nar/gku1280 (2015).

2 Consortium, E. P. The ENCODE (ENCyclopedia Of DNA Elements) Project. *Science* **306**, 636-640, doi:10.1126/science.1105136 (2004).

3 Gu, J. *et al.* Mapping of Variable DNA Methylation Across Multiple Cell Types Defines a Dynamic Regulatory Landscape of the Human Genome. *G3 (Bethesda)* **6**, 973-986, doi:10.1534/g3.115.025437 (2016).

4 Bonder, M. J. *et al.* Disease variants alter transcription factor levels and methylation of their binding sites. *Nat Genet* **49**, 131-138, doi:10.1038/ng.3721 (2017).

5 Joubert, B. R. *et al.* DNA Methylation in Newborns and Maternal Smoking in Pregnancy: Genome-wide Consortium Meta-analysis. *Am J Hum Genet* **98**, 680-696, doi:10.1016/j.ajhg.2016.02.019 (2016).

6 Sharp, G. C. *et al.* Maternal BMI at the start of pregnancy and offspring epigenome-wide DNA methylation: findings from the pregnancy and childhood epigenetics (PACE) consortium. *Hum Mol Genet* **26**, 4067-4085, doi:10.1093/hmg/ddx290 (2017).

7 Smith, A. K. *et al.* Methylation quantitative trait loci (meQTLs) are consistently detected across ancestry, developmental stage, and tissue type. *BMC Genomics* **15**, 145, doi:10.1186/1471-2164-15-145 (2014).

8 Wagner, J. R. *et al.* The relationship between DNA methylation, genetic and expression inter-individual variation in untransformed human fibroblasts. *Genome Biol* **15**, R37, doi:10.1186/gb-2014-15-2-r37 (2014).

9 Teh, A. L. *et al.* The effect of genotype and in utero environment on interindividual variation in neonate DNA methylomes. *Genome Res* **24**, 1064-1074, doi:10.1101/gr.171439.113 (2014).

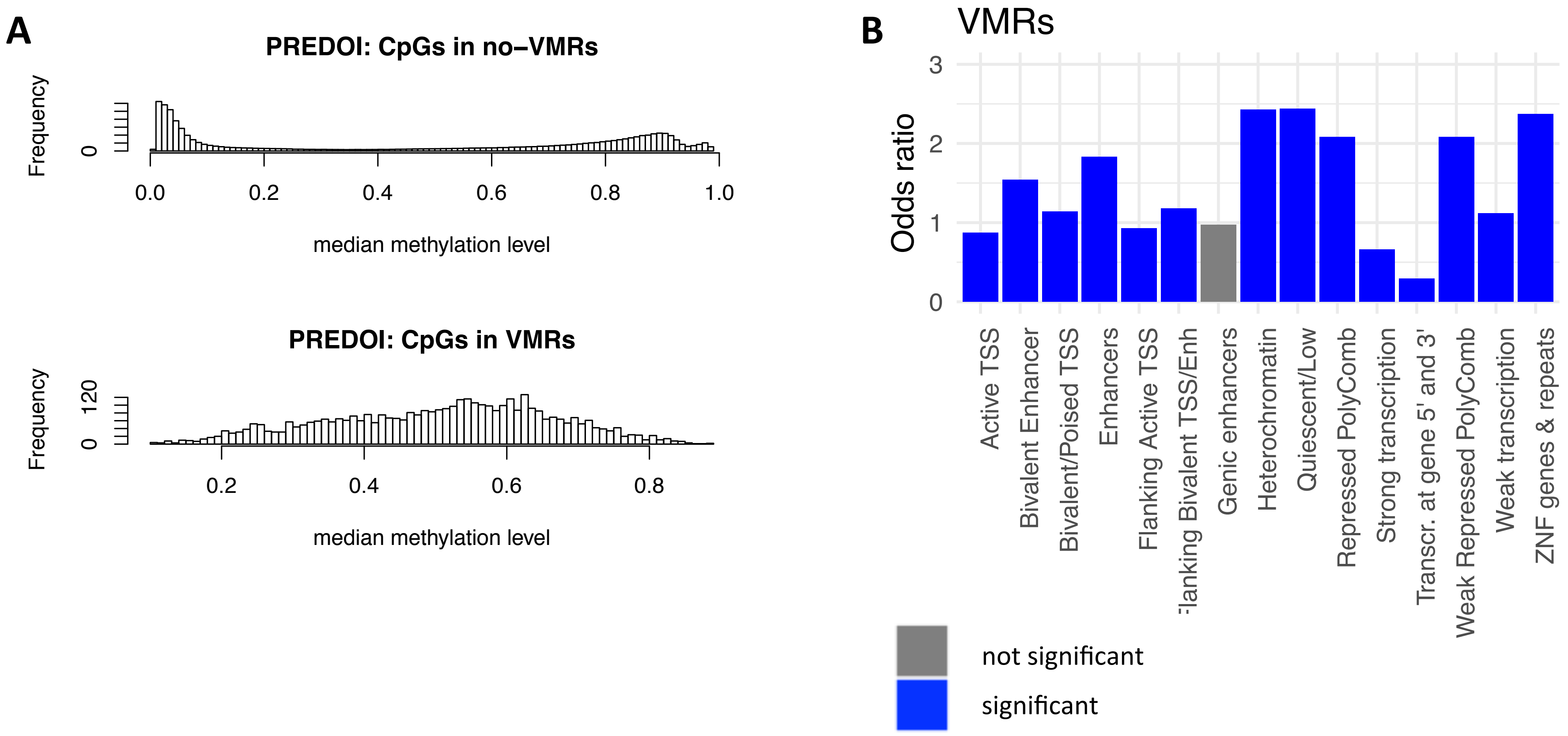

Supplementary Figure 1: Distribution of VMRs

A: Histogram of median methylation levels of PREDO I for CpG-sites located in non-VMRs (above) and CpG-sites located in VMRs (below).
B: VMR enrichment for histone marks. Significant enrichment/depletion is depicted in blue, non-significance in grey, based on Fisher-tests.

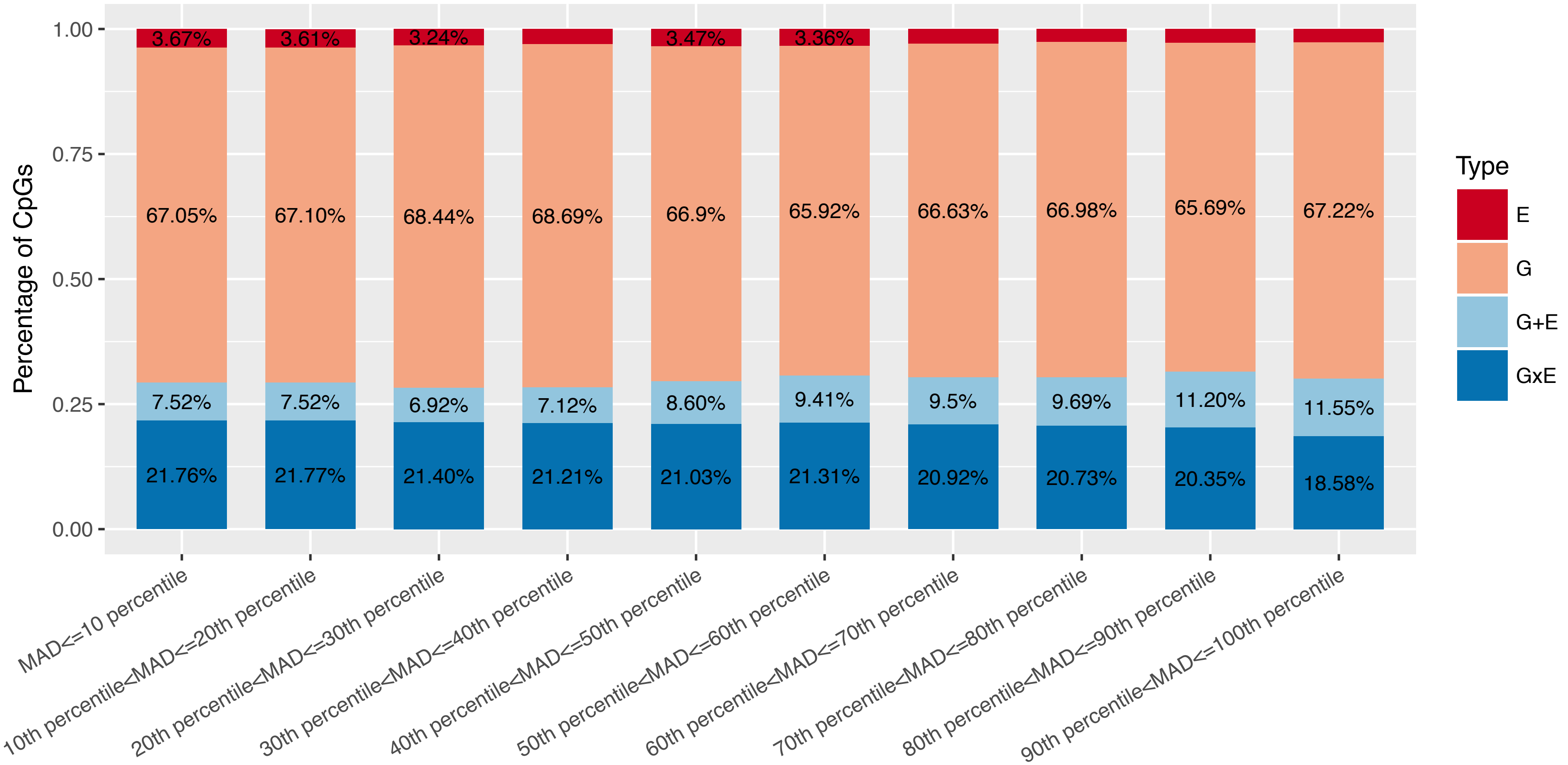

Supplementary Figure 2: Distribution of best models stratified by CpG variability

Percentage of models (G, maternal age, Gxmaternal age or G+maternal age) with the lowest AIC explaining variable DNA methylation in PREDO I. The different columns indicate which percentiles of the MAD-score were used to select the CpGs.

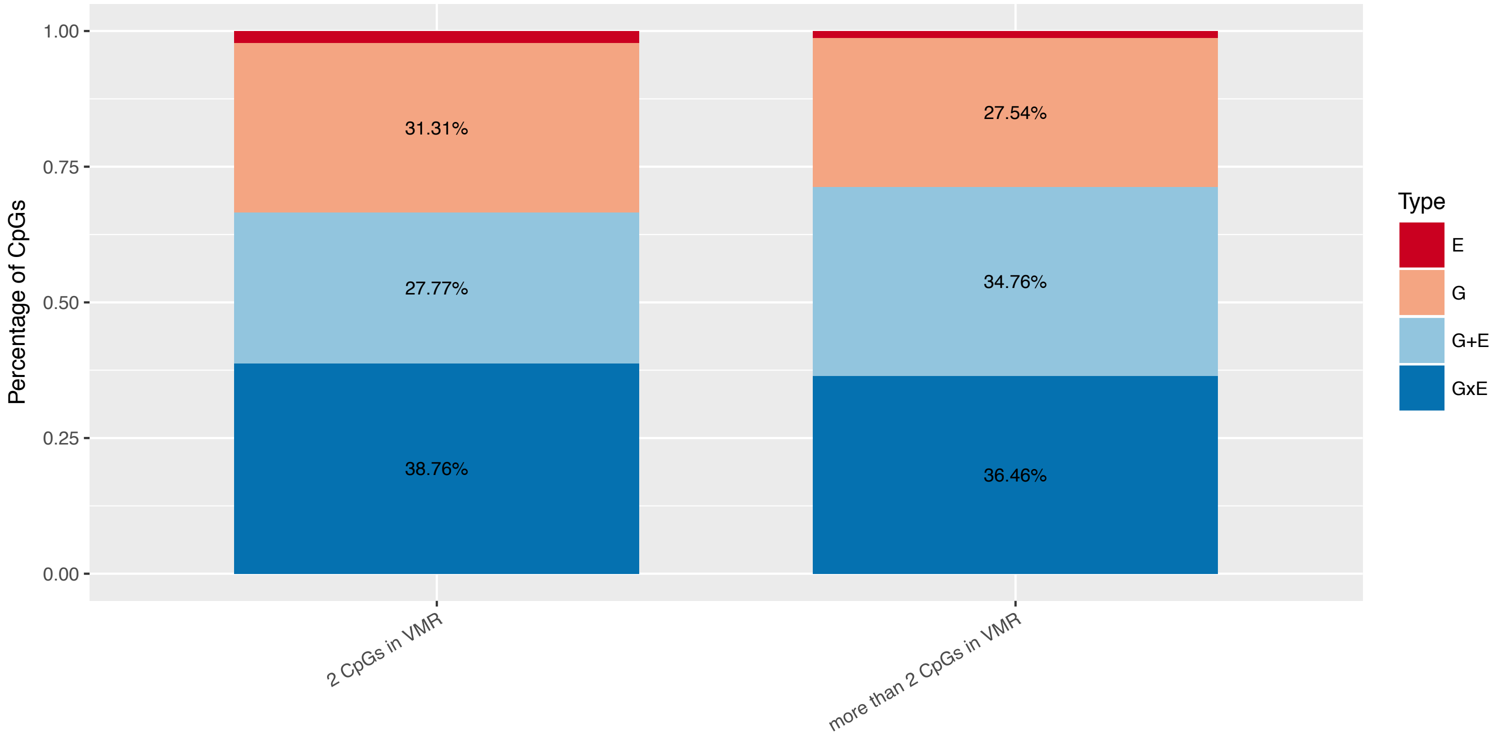

Supplementary Figure 3: Distribution of best models stratified by VMR length

Distribution of best models in PREDO I with regard to VMRs including 2 CpGs (left) and VMRs including at least 3 CpGs (right).

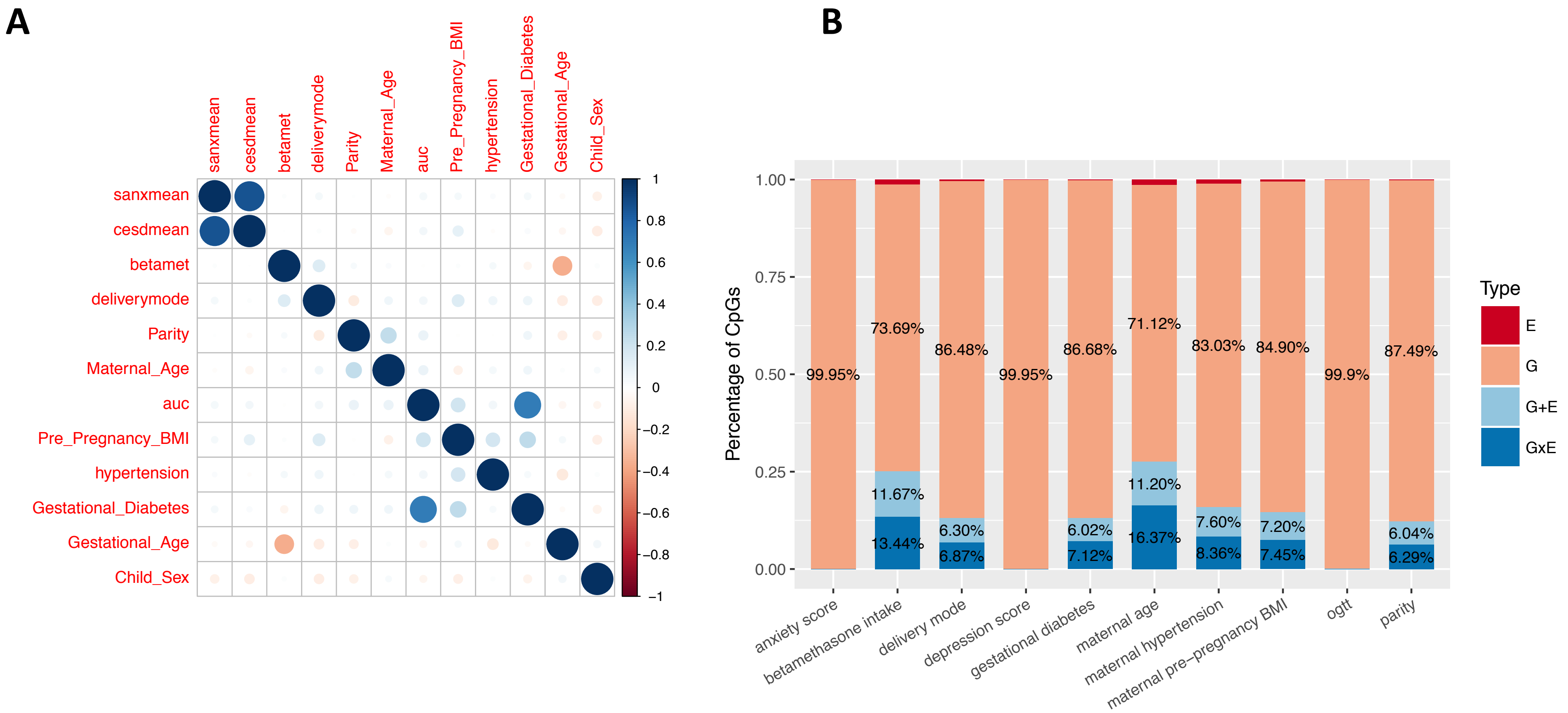

Supplementary Figure 4: Correlation of prenatal phenotypes and distribution of best models stratified by prenatal environment

A: correlation plot of environmental phenotypes in PREDO I. Larger circles represent higher absolute correlation values.
B: percentage of best models in PREDO I, stratified by E.

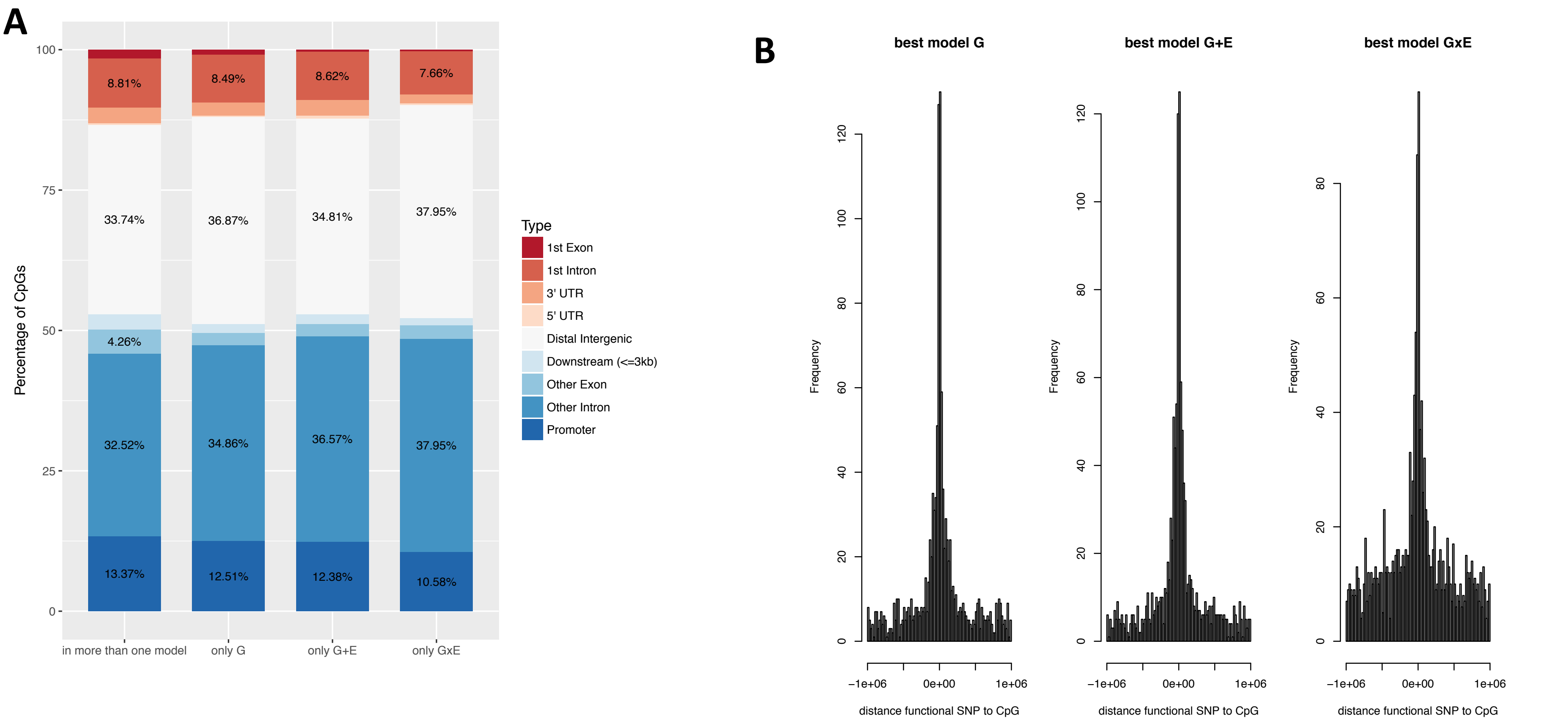

Supplementary Figure 5: Mapping of DeepSEA variants involved in best model G, G+E and GxE

A: Location of all of DeepSEA variants involved in best model G, G+E and GxE on the 450k array in relationship to CpG-Islands using the Illumina 450K annotation.
To increase readability all counts < 3% have been omitted.
B: Distance between the respective DeepSEA variant and tagCpG within best model G (left), best model G+E (middle) and best model GxE (right). The X-axis denotes the distance in bases between the SNPs and the tagCpG, the Y-axis the frequency of SNP/VMR pairs with this distance.

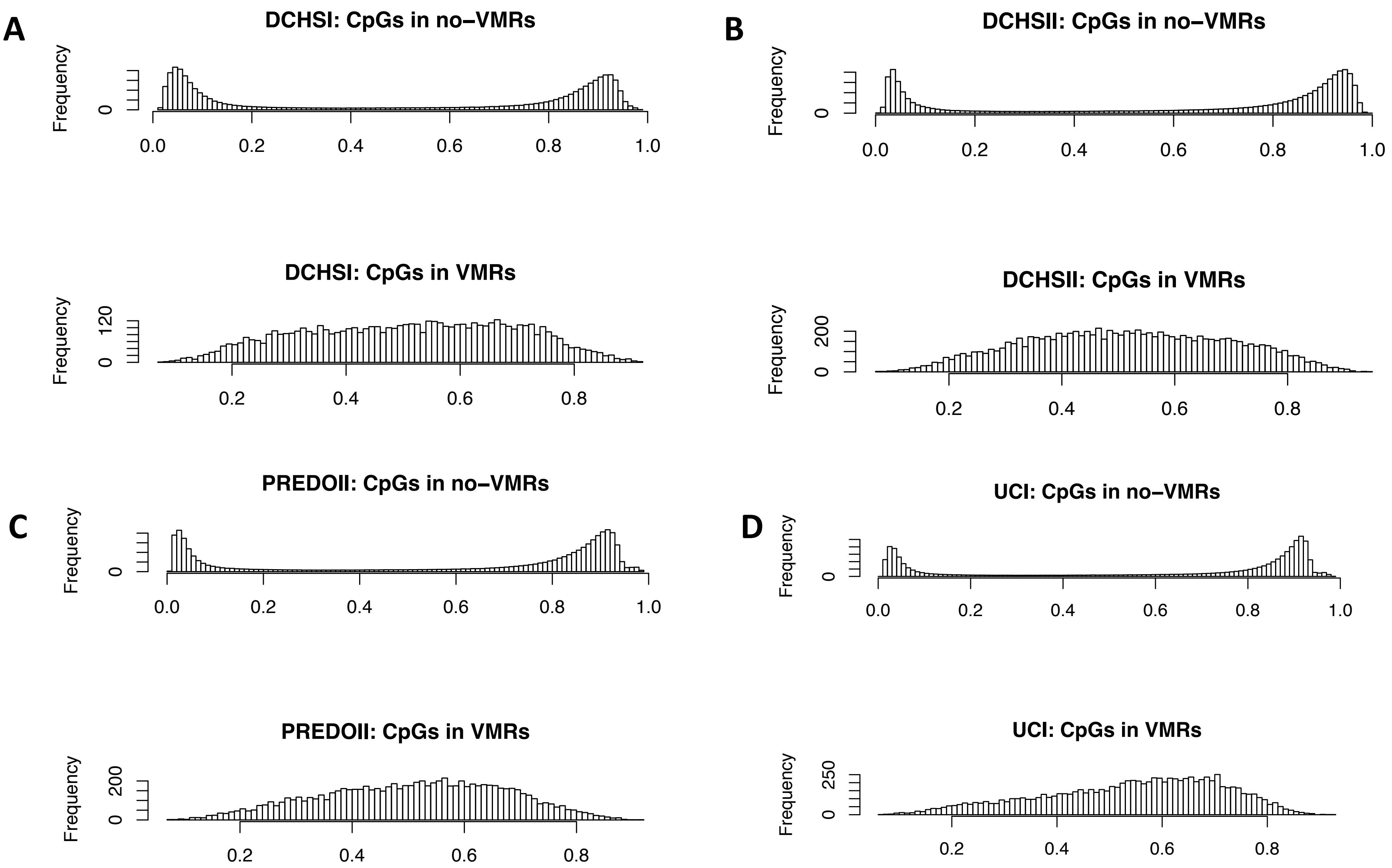

Supplementary Figure 6: Distribution of CpGs in VMRs across all cohorts

Histogram of median methylation levels of CpG-sites located with in non-VMRs (above) and within VMRs (below) for DCHS I (A), DCHS II (B), PREDO II (C) and UCI (D).

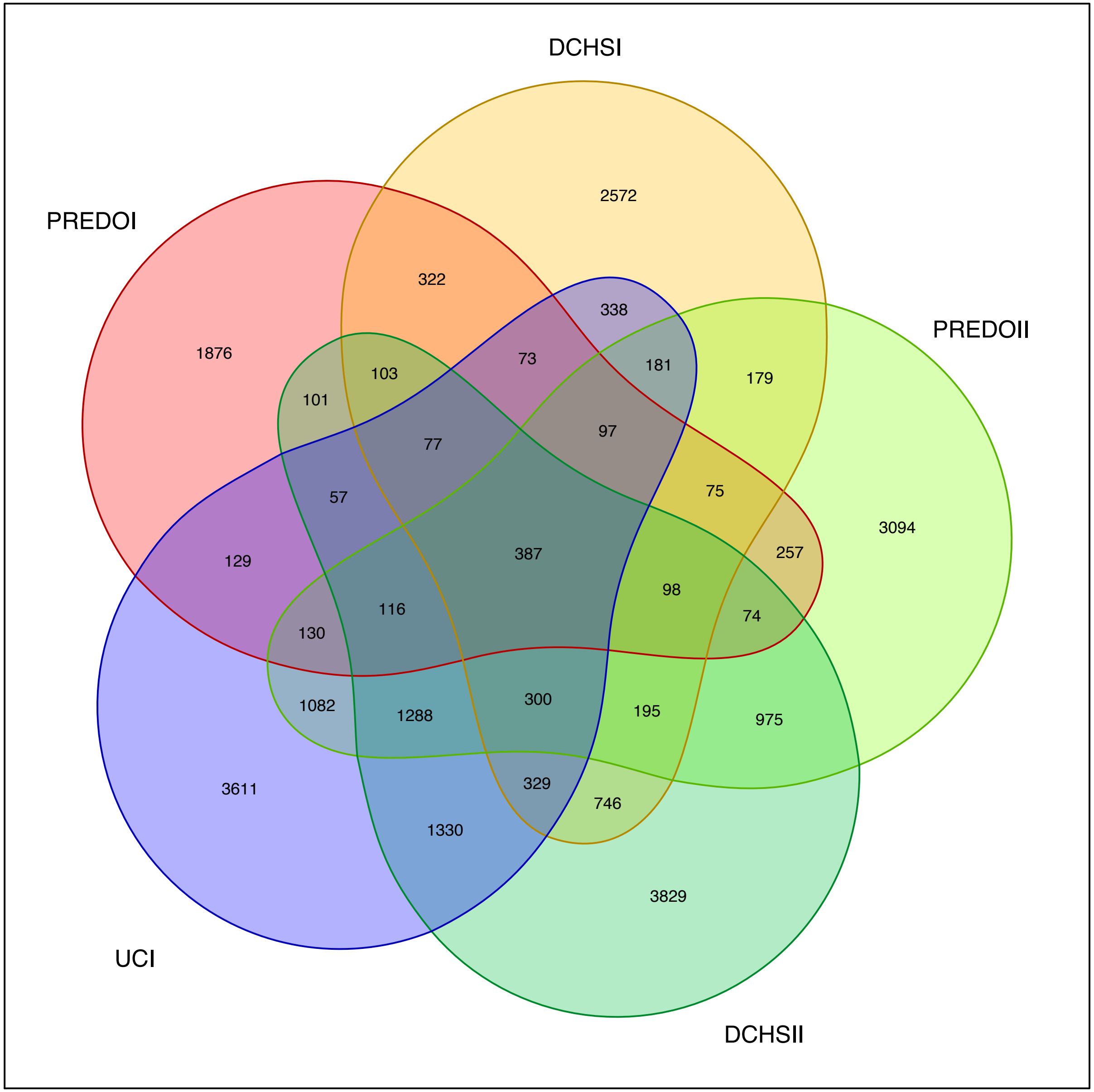

Supplementary Figure 7: Venn-diagram of overlapping tagCpgs

Venn-diagram of overlapping tagCpGs for PREDO I, PREDO II, DCHS I, DCHS II and UCI

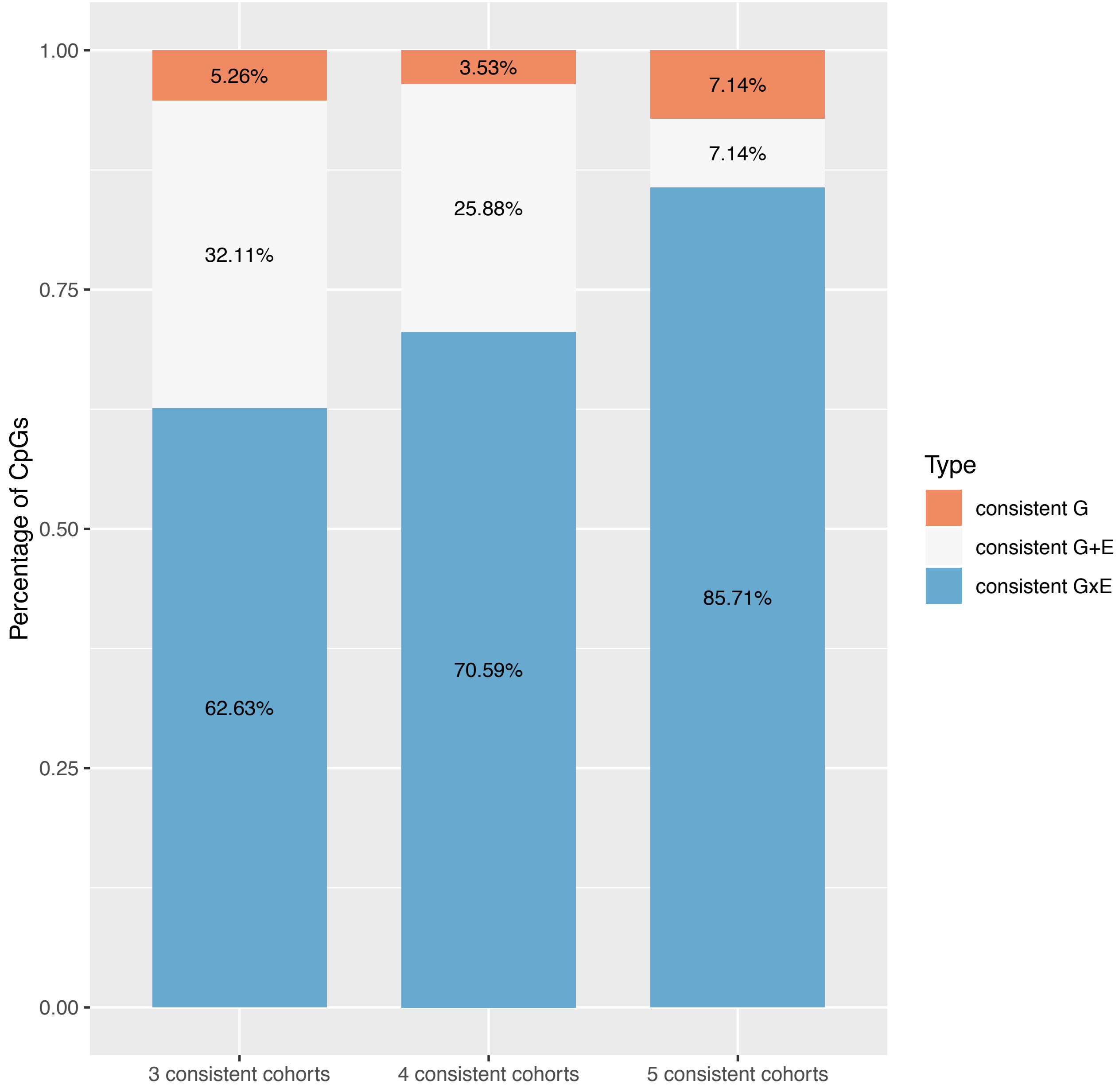

Supplementary Figure 8: Consistency of best models across all cohorts

Consistency of best models across overlapping tagCpgs

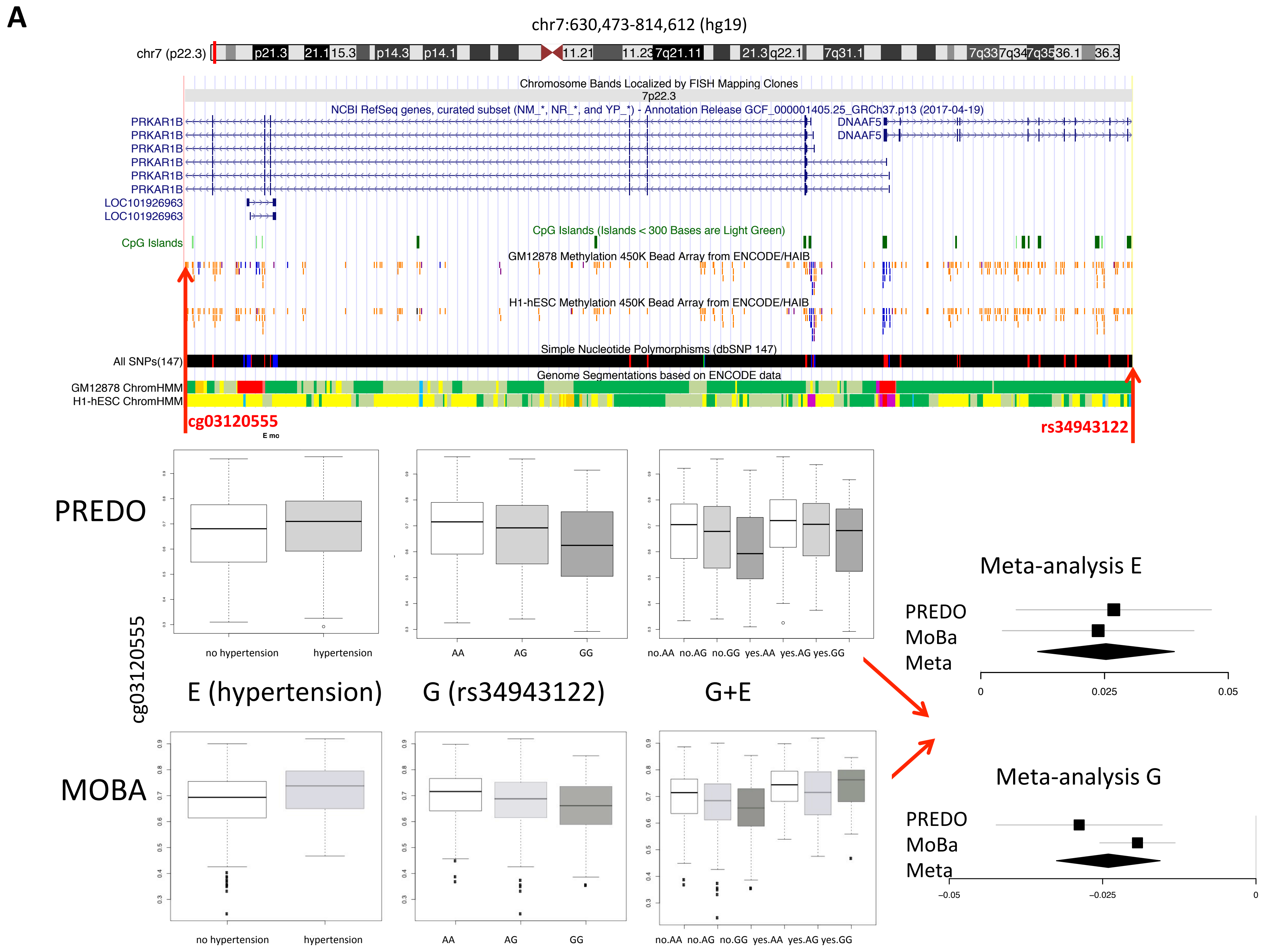
A
chr7:630,473-814,612 (hg19)
chr7 (p22.3)
Chromosome Bands Localized by FISH Mapping Clones
7p22.3
NCBI RefSeq genes, curated subset (NM_*, NR_*, and YP_*) - Annotation Release GCF_000001405.25_GRCh37.p13 (2017-04-19)
PRKAR1B
DNAAF5
LOC101926963
CpG Islands
CpG Islands (Islands < 300 Bases are Light Green)
GM12878 Methylation 450K Bead Array from ENCODE/HAIB
H1-hESC Methylation 450K Bead Array from ENCODE/HAIB
All SNPs(147)
Simple Nucleotide Polymorphisms (dbSNP 147)
Genome Segmentations based on ENCODE data
GM12878 ChromHMM
H1-hESC ChromHMM
cg03120555
rs34943122
PREDO
MOBA
cg03120555
no hypertension
hypertension
AA
AG
GG
no.AA no.AG no.GG yes.AA yes.AG yes.GG
E (hypertension)
G (rs34943122)
G+E
Meta-analysis E
PREDO
MoBa
Meta
0
0.025
0.05
Meta-analysis G
PREDO
MoBa
Meta
−0.05
−0.025
0

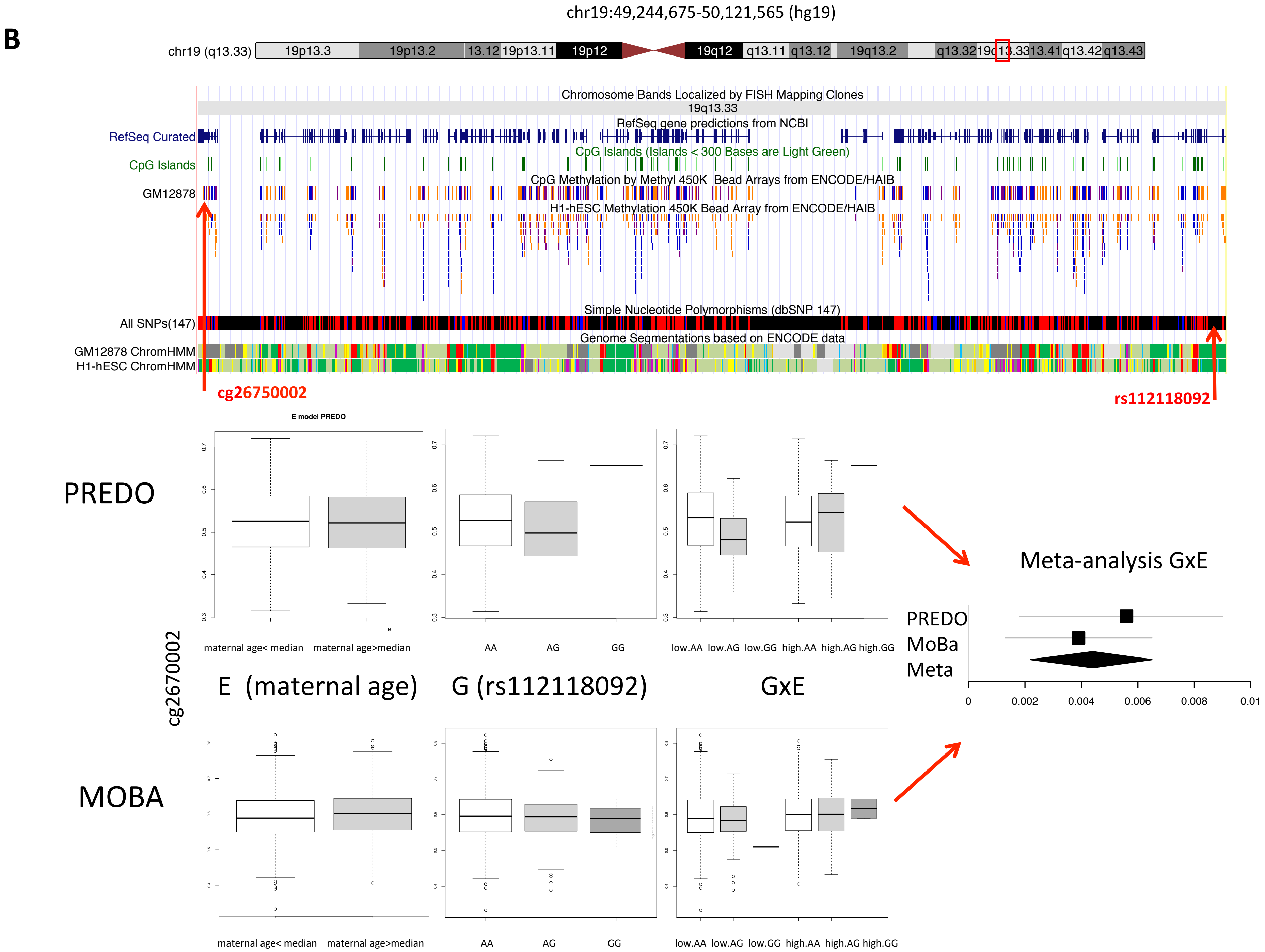
B
chr19:49,244,675-50,121,565 (hg19)
chr19 (q13.33)
19p13.3
19p13.2
13.12
19p13.11
19p12
19q12
q13.11
q13.12
19q13.2
q13.32
19q13.33
13.41
q13.42
q13.43
Chromosome Bands Localized by FISH Mapping Clones
19q13.33
RefSeq gene predictions from NCBI
RefSeq Curated
CpG Islands (Islands < 300 Bases are Light Green)
CpG Islands
CpG Methylation by Methyl 450K Bead Arrays from ENCODE/HAIB
GM12878
H1-hESC Methylation 450K Bead Array from ENCODE/HAIB
Simple Nucleotide Polymorphisms (dbSNP 147)
All SNPs(147)
Genome Segmentations based on ENCODE data
GM12878 ChromHMM
H1-hESC ChromHMM
cg26750002
rs112118092
E model PREDO
PREDO
maternal age< median
maternal age>median
AA
AG
GG
low.AA
low.AG
low.GG
high.AA
high.AG
high.GG
cg2670002
E (maternal age)
G (rs112118092)
GxE
Meta-analysis GxE
PREDO
MoBa
Meta
0
0.002
0.004
0.006
0.008
0.01
MOBA
maternal age< median
maternal age>median
AA
AG
GG
low.AA
low.AG
low.GG
high.AA
high.AG
high.GG

Supplemental Figure 9: Top hits of meta-analysis of PREDO I and MoBa

Illustration of top hits of meta-analysis. SNPs and CpGs are depicted relative to their genomic location and the following UCSC tracks (based on gh19): NCBI RefSeq genes, CpG Islands, GM12878 Methylation 450k BeadArray from ENCODE/HAIB, H1-hESC Methylation 450k BeadArray from ENCODE/HAIB, simple nucleotide polymorphism (db SNP147), genome segmentations based on ENCODE data for GM12878, genome segmentations based on ENCODE data for H1-hESC. In the lower part of the panel, boxplots are given for the results from PREDO (upper panels) and MoBa (lower panels). Y-axis denotes the respective beta-values and the X-axis the different environmental conditions or genotypes. The median is depicted by a black line, the rectangle spans the first quartile to the third quartile, whiskers above and below the box show the location of minimum and maximum beta-values. On the right side, a forest-plot is given where the effect size estimate is depicted as black square and the grey line indicates the respective confidence interval on the X-axis. The Y-axis denotes the different studies and the meta-analysis. The result of the meta-analysis is depicted as a diamond: the center line of the diamond gives the effect size estimator from the meta-analysis while the lateral tips of the diamond indicated the lower and upper limits of the confidence interval.

A: Illustration of one of the top G+E hits from the meta-analysis, including location of SNP rs34943122 and CpG cg03120555. Cg03120555 is located in *PRKAR1B*, a gene which has been associated with dementia and is related to signaling by Hedgehog, rs34943122 is located in *HEATR2,* which has been associated with Primary Ciliary Dyskinesia.

B: Illustration of one of the top GxE hits from the meta-analysis, including location of SNP rs112118092 and CpG cg26750002. Cg26750002 is located in IZUMO1 which is essential for sperm-egg plasma-membrane binding and function. rs112118092 is located in *PRR12,* which has been linked to intellectual disability and neuropsychiatric problems.

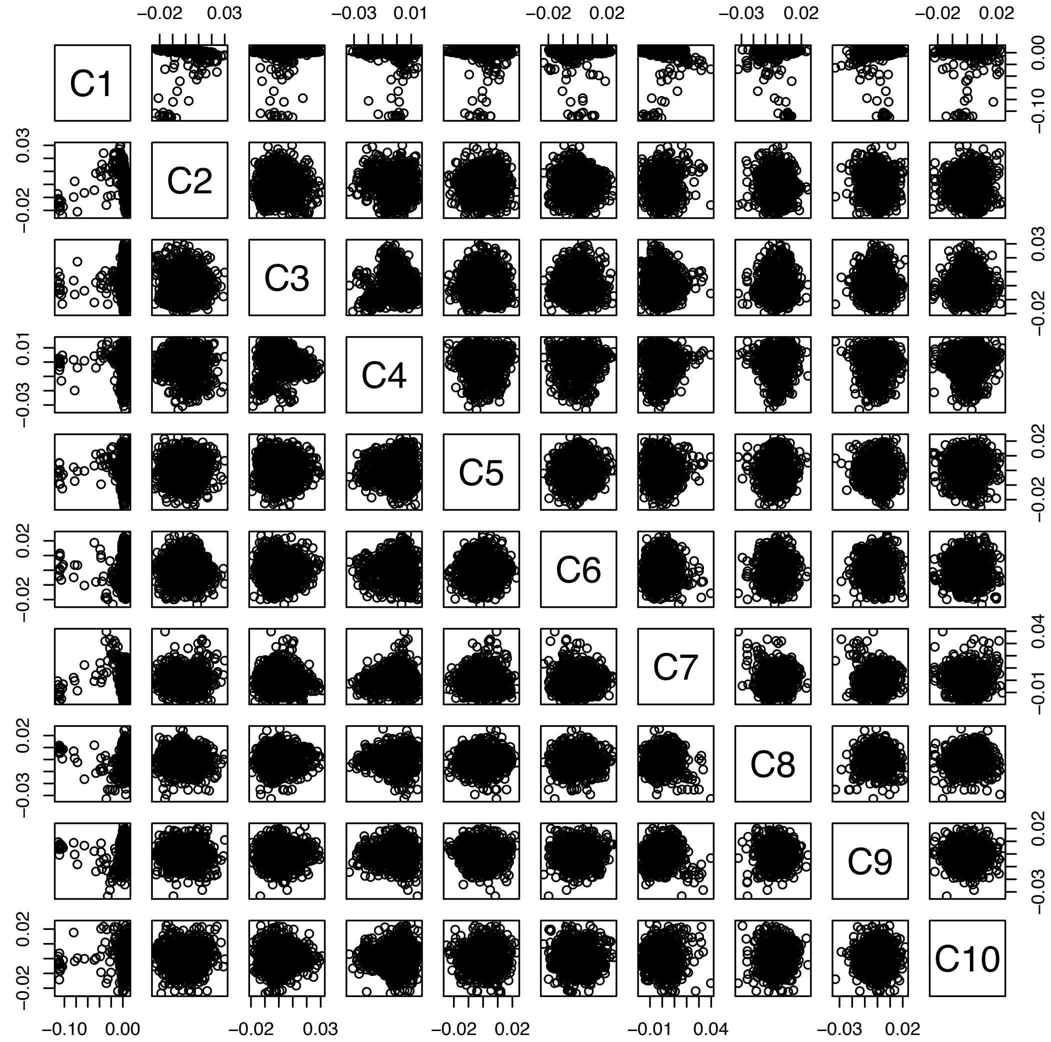

Supplementary Figure 10: MDS-plot of PREDO

MDS (multi-dimensional-scaling)-plot of the first ten components of the MDS-analysis on the IBD (identical-by-state) matrix for PREDO. The first two components reflect the subcluster of individuals with mixed ancestry.
