# Supplementary material for: Integrated analysis of environmental and genetic influences on cord blood DNA methylation in new-borns
Source: Nat Commun. 2019 Jun 11;10:2548. doi: 10.1038/s41467-019-10461-0 (PMC6559955; doi:10.1038/s41467-019-10461-0)
Supplement: Supplementary file 3 — Description of Additional Supplementary Files [file 41467_2019_10461_MOESM3_ESM.docx]

**Description of Supplementary Files**

**File Name:** **Supplementary Data 1.**

**Description:** Identified VMRs in PREDO I all CpGs located in VMRs are depicted MAD: median absolute deviation bp_CpG: genomic position of CpG in base-pairs (hg19) Relation to Island is given according to Illumina 450k annotation: Island, N_Shore (north shore), N_Shelf (north shelf), S_Shore (south shore), S_Shelf (south shelf), OpenSea genes are given according to Illumina 450K annotation.

**File Name: Supplementary Data 2.**

**Description:** Nominal significant results for model G in pruned PREDO I dataset only the SNP with the lowest AIC for the respective tagCpG is displayed bp_CpG: genomic position of CpG in base-pairs (hg19) bp_SNP: genomic position of SNP in base-pairs (hg19) AIC_SNP: AIC of specific G model num_snps in cis window: number of SNPs in the respective cis window which were tested for in the pruned data set p_SNP: nominal p-value of G effect p_SNP_corrected_cis_window: p-value of G effect corrected for the number of tested SNPs in the specific cis window (based on Bonferroni-correction) p_SNP-corrected_overall: FDR corrected p-value of G effect (corrected for the number of tested SNPs in the specific cis window and corrected for all tested tagCpGs).

**File Name:** **Supplementary Data 3.**

**Description:** Nominal significant results for model E in pruned PREDO I dataset only the E with the lowest AIC for the respective tagCpG is displayed bp_CpG: genomic position of CpG in base-pairs (hg19) best_E: environment giving the lowest AIC AIC_E: AIC of specific E model p_E: nominal p-value of E effect p_E_corrected_env: p-value of E effect corrected for all tested environmental phenotypes (based on Bonferroni-correction) p_E_corrected_overall: FDR-corrected p-value of E effect (corrected for all tested environmental phenotypes and corrected for all tested tagCpGs).

**File Name:** **Supplementary Data 4.**

**Description:** Nominal significant association results for model G+E in pruned PREDO I dataset only the SNP-environment combinations with the lowest AIC for the respective tagCpG are displayed bp_CpG: genomic position of CpG in base-pairs (hg19) SNP_add: SNP included in G+E model bp_SNP: genomic position of SNP in base-pairs (hg19) E_add: environment included in G+E model AIC_add: AIC of specific G+E model num_snps in cis window: number of SNPs in the respective cis window which were tested for in the pruned data set p_add: nominal p-value of G+E model (based on ANOVA of model G and model G+E) p_add_corrected_combinations: p-value of G+E model corrected for all tested SNP-environment combinations in cis-window (based on Bonferroni-correction) p_add_corrected_overall: FDR-corrected p-value of G+E model (corrected for all tested SNP-environment combinations in cis-window and corrected for all tested tagCpGs).

**File Name:** **Supplementary Data 5.**

**Description:** Nominal significant association results for model GxE in pruned PREDO I dataset only the SNP-environment combinations with the lowest AIC for the respective tagCpG are displayed bp_CpG: genomic position of CpG in base-pairs (hg19) SNP_int: SNP included in GxE model bp_SNP: genomic position of SNP in base-pairs (hg19) E_int: environment included in GxE model AIC_int: AIC of specific GxE model num_snps in cis window: number of SNPs in the respective cis window which were tested for in the pruned data set p_int: nominal p-value for GxE effect p_int_corrected_combinations: p-value of GxE model corrected for all tested SNP-environment combinations in cis-window (based on Bonferroni-correction) p_int_corrected_overall: FDR-corrected p-value of GxE model (corrected for all tested SNP-environment combinations in cis-window and corrected for all tested tagCpGs).

**File Name:** **Supplementary Data 6.**

**Description:** tagCpGs with best model G in pruned PREDO I dataset bp_CpG: genomic position of CpG in base-pairs (hg19) SNP: SNP included in G model bp_SNP: genomic position of SNP in base-pairs (hg19) AIC_SNP: AIC of specific G model p_SNP: nominal p-value of G effect.

**File Name:** **Supplementary Data 7.**

**Description:** tagCpGs with best model G+E in pruned PREDO I dataset bp_CpG: genomic position of CpG in base-pairs (hg19) SNP_add: SNP included in G+E model bp_SNP: genomic position of SNP in base-pairs (hg19) E_add: E included in G+E model AIC_add: AIC of specific G+E model p_add: nominal p-value of G+E model (based on ANOVA of model G and model G+E).

**File Name:** **Supplementary Data 8**

**Description:** tagCpGs with best model GxE in pruned PREDO I dataset bp_CpG: genomic position of CpG in base-pairs (hg19) SNP_int: SNP included in GxE model bp_SNP: genomic position of SNP in base-pairs (hg19) E_int: E included in GxE model AIC_int: AIC of specific GxE model p_int: nominal p-value of GxE effect.

**File Name:** **Supplementary Data 9**

**Description:** nominal significant results for CpGs for model G using DeepSEA variants in PREDO I only the SNP with the lowest AIC for the respective tagCpG is displayed bp_CpG: genomic position of CpG in base-pairs (hg19) bp_SNP: genomic position of SNP in base-pairs (hg19) AIC_SNP: AIC of specific G model num_snps in cis window: number of deepSEA SNPs in the respective cis window which were tested for p_SNP: nominal p-value of G effect p_SNP_corrected_cis_window: p-value of G effect corrected for the number of tested SNPs in the specific cis window (based on Bonferroni-correction) p_SNP-corrected_overall: FDR corrected p-value of G effect (corrected for the number of tested SNPs in the specific cis window and corrected for all tested tagCpGs).

**File Name:** **Supplementary Data 10.**

**Description:** nominal significant results for model E using DeepSEA variants in PREDO I only the E with the lowest AIC for the respective tagCpG is displayed bp_CpG: genomic position of CpG in base-pairs (hg19) best_E: environment giving the lowest AIC AIC_E: AIC of specific E model p_E: nominal p-value of E effect p_E_corrected_env: p-value of E effect corrected for all tested environmental phenotypes (based on Bonferroni-correction) p_E_corrected_overall: FDR-corrected p-value of E effect (corrected for all tested environmental phenotypes and corrected for all tested tagCpGs).

**File Name:** **Supplementary Data 11.**

**Description:** nominal significant results for model G+E using DeepSEA variants in PREDO I only the SNP-environment combinations with the lowest AIC for the respective tagCpG are displayed bp_CpG: genomic position of CpG in base-pairs (hg19) SNP_add: SNP included in G+E model bp_SNP: genomic position of SNP in base-pairs (hg19) E_add: environment included in G+E model AIC_add: AIC of specific G+E model num_snps in cis window: number of deepSEA SNPs in the respective cis window which were tested for p_add: nominal p-value of G+E model (based on ANOVA of model G and model G+E) p_add_corrected_combinations: p-value of G+E model corrected for all tested SNP-environment combinations in cis-window (based on Bonferroni-correction) _add_corrected_overall: FDR-corrected p-value of G+E model (corrected for all tested SNP-environment combinations in cis-window and corrected for all tested tagCpGs).

**File Name:** **Supplementary Data 12.**

**Description:** nominal significant results for model GxE using DeepSEA variants in PREDO I only the SNP-environment combinations with the lowest AIC for the respective tagCpG are displayed bp_CpG: genomic position of CpG in base-pairs (hg19) SNP_int: SNP included in GxE model bp_SNP: genomic position of SNP in base-pairs E_int: environment included in GxE model (hg19) AIC_int: AIC of specific GxE model num_funct_snps in cis window: number of DeepSEA SNPs in the respective cis window which were tested for p_int: nominal p-value for GxE effect p_int_corrected_combinations: p-value of GxE model corrected for all tested SNP-environment combinations in cis-window (based on Bonferroni-correction) p_int_corrected_overall: FDR-corrected p-value of GxE model (corrected for all tested SNP-environment combinations in cis-window and corrected for all tested tagCpGs).

**File Name:** **Supplementary Data 13.**

**Description:** tagCpGs with best model G using DeepSEA variants in PREDO I bp_CpG: genomic position of CpG in base-pairs (hg19) SNP: SNP included in G model bp_SNP: genomic position of SNP in base-pairs (hg19) AIC_SNP: AIC of specific G model p_SNP: nominal p-value of G effect.

**File Name:** **Supplementary Data 14.**

**Description:** tagCpGs with best model E using DeepSEA variants in PREDO I bp_CpG: genomic position of CpG in base-pairs (hg19) E: environment included in E model AIC_E: AIC of E model p_E: nominal p-value of E effect.

**File Name:** **Supplementary Data 15.**

**Description:** tagCpGs with best model G+E using DeepSEA variants in PREDO I bp_CpG: genomic position of CpG in base-pairs (hg19) SNP_add: SNP included in G+E model bp_SNP: genomic position of SNP in base-pairs (hg19) E_add: E included in G+E model AIC_add: AIC of specific G+E model p_add: nominal p-value of G+E model (based on ANOVA of model G and model G+E)

**File Name:** **Supplementary Data 16.**

**Description:** tagCpGs with best model GxE using DeepSEA variants in PREDO I bp_CpG: genomic position of CpG in base-pairs (hg19) SNP_int: SNP included in GxE model bp_SNP: genomic position of SNP in base-pairs (hg19) E_int: E included in GxE model AIC_int: AIC of specific GxE model p_int: nominal p-value of GxE effect.

**File Name:** **Supplementary Data 17.**

**Description:** top-results of meta-analysis of PREDO and MoBa for G+E model bp_CpG: genomic position of CpG in base-pairs SNP_add: SNP included in G+E model bp_SNP: genomic position of SNP in base-pairs E_add: E included in G+E model MAF_PREDO: minor allele frequency in PREDO beta_add_SNP_PREDO: effect size estimator for main G effect in PREDO p_add_SNP_PREDO: nominal p-value of G effect in G+E model in PREDO beta_add_E_PREDO: effect size estimator for main E effect in PREDO p_add_E_PREDO: nominal p-value of E effect in G+E model in PREDO MAF_MoBa: minor allele frequency in MoBa beta_add_SNP_MoBa: effect size estimator for main G effect in MoBa p_add_SNP_MoBa: nominal p-value of G effect in G+E model in MoBa beta_add_E_MoBa: effect size estimator for main E effect in MoBa p_add_E_MoBa: nominal p-value of E effect in G+E model in MoBa beta_add_SNP_meta: effect size estimator for main G effect in meta-analysis p_add_SNP_meta: nominal p-value for G effect in meta-analysis p_add_SNP_meta_FDR: FDR corrected p-value for G effect in meta-analysis across all tested SNP-E combinations beta_add_E_meta: effect size estimator for main E effect in meta-analysis p_add_E_meta: nominal p-value for E effect in meta-analysis p_add_E_meta_FDR: FDR corrected p-value for E effect in meta-analysis across all tested SNP-E combination.

**File Name:** **Supplementary Data 18.**

**Description:** top-results of meta-analysis of PREDO and MoBa for GxE model bp_CpG: genomic position of CpG in base-pairs SNP_int: SNP included in G+E model bp_SNP: genomic position of SNP in base-pairs E_int: E included in G+E model MAF_PREDO: minor allele frequency in PREDO beta_int_PREDO: effect size estimator for GxE effect in PREDO p_int_PREDO: nominal p-value for GxE in PREDO MAF_MoBa: minor allele frequency in MoBa beta_int_MoBa: effect size estimator for GxE effect in MoBa p_int_MoBa: nominal p-value for GxE in MoBa beta_int_meta: effect size estimator for GxEt in meta-analysis p_int_meta: p-value for GxE in meta-analysis p_int_meta_FDR: FDR-corrected p-value for GxE in meta-analysis across all tested SNP-E combinations.
